# Cost-Effectiveness of Dapagliflozin versus Acarbose as a Monotherapy in Type 2 Diabetes in China

**DOI:** 10.1371/journal.pone.0165629

**Published:** 2016-11-02

**Authors:** Shuyan Gu, Yiming Mu, Suodi Zhai, Yuhang Zeng, Xuemei Zhen, Hengjin Dong

**Affiliations:** 1 Center for Health Policy Studies, School of Public Health, Zhejiang University School of Medicine, Hangzhou City, Zhejiang, China; 2 Department of Endocrinology and Metabolism, Chinese PLA General Hospital, Chinese PLA Medical College, Beijing, China; 3 Department of Pharmacy, Peking University Third Hospital, Beijing, China; Shanghai Diabetes Institute, CHINA

## Abstract

**Objective:**

To estimate the long-term cost-effectiveness of dapagliflozin versus acarbose as monotherapy in treatment-naïve patients with type 2 diabetes mellitus (T2DM) in China.

**Methods:**

The Cardiff Diabetes Model, an economic model designed to evaluate the cost-effectiveness of comparator therapies in diabetes was used to simulate disease progression and estimate the long-term effect of treatments on patients. Systematic literature reviews, hospital surveys, meta-analysis and indirect treatment comparison were conducted to obtain model-required patient profiles, clinical data and costs. Health insurance costs (2015¥) were estimated over 40 years from a healthcare payer perspective. Univariate and probabilistic sensitivity analyses were performed.

**Results:**

The model predicted that dapagliflozin had lower incidences of cardiovascular events, hypoglycemia and mortality events, was associated with a mean incremental benefit of 0.25 quality-adjusted life-years (QALYs) and with a lower cost of ¥8,439 compared with acarbose. This resulted in a cost saving of ¥33,786 per QALY gained with dapagliflozin. Sensitivity analyses determined that the results are robust.

**Conclusion:**

Dapagliflozin is dominant compared with acarbose as monotherapy for Chinese T2DM patients, with a little QALY gain and lower costs. Dapagliflozin offers a well-tolerated and cost-effective alternative medication for treatment-naive patients in China, and may have a direct impact in reducing the disease burden of T2DM.

## Introduction

Diabetes as one of the most threatening noncommunicable diseases, imposes great health challenges and heavy disease burden on patients and healthcare systems [1]. The disease burden of diabetes is escalating in China, the International Diabetes Federation (IDF) reports that diabetes prevalence in people aged 20–79 years is at 10.6% in 2015 with China having the highest number of diabetics (109.6 million) worldwide [2]. However, glycaemic control remains elusive for the majority of Chinese diabetics. Only 25.8% of patients receive diabetes-related treatments; and only 39.7% of those treated have adequate glycaemic control [3]. Mortality in diabetes is high, with 1.3 million diabetes-related deaths in 2015 [2] with type 2 diabetes mellitus (T2DM) accounting for at least 90% of these cases [4]. Correspondingly, diabetes-related health expenditure in China is high (51 billion US dollars) in 2015, ranked second worldwide [2].

Long-term glycaemic control is fundamental in the management of T2DM [5–8], where a patient-centered treatment strategy needs to comprehensively consider patient’s characteristics, comorbidities and optimize for efficacy, tolerability, safety and costs [9–10]. The excessive mortality and costs associated with diabetes are largely attributable to diabetes-related complications and metabolic risk factors including high blood pressure, overweight and obesity [11–14]. However, hypertension and obesity are common comorbidities of diabetes [1]. Over 75% of patients with diabetes have systolic blood pressure (SBP) levels of ≥140/80 mmHg or are taking antihypertensive medications simultaneously [15]. In China, 72% of patients reported either comorbid hypertension, dyslipidemia, or both [16]. Moreover, being overweight or obese is also common in patients with diabetes (85%) and is usually related to hypertension and dyslipidemia [1,17]. These risk factors may increase the risk of macrovascular and microvascular disease for patients [1,16,18]. On the other hand, treatment-induced adverse effects such as hypoglycaemia and weight gain are commonly observed in many of available medications [1,9,19], which may impede treatment effect by suboptimal dosing and/or poor medication adherence; thus increase the risks of diabetes-related complications and elevate treatment costs [20–24]. Optimization of diabetes treatment requires a multi-factorial approach which goes beyond blood glucose control and encompasses risk factors including reduction of hypoglycaemia risk, body weight and blood pressure, so as to reduce disease burden [1,6,16,25]. However, only 13.7% of patients with T2DM achieved blood glucose and blood pressure control, and only 5.6% achieved blood glucose, blood pressure and blood lipids control in China [16]. Thus there is an unmet medical need for novel effective agents with improved risk profiles and minimized adverse effects.

Dapagliflozin is a novel, competitive and selective oral sodium glucose cotransporter 2 (SGLT2) inhibitor. It lowers blood glucose independent of insulin secretion and acts by inhibiting renal glucose reabsorption, thus promoting increased urinary glucose excretion and calories loss [26–27]. Therefore, dapagliflozin may produce consistent and durable glycaemic control at any stage of insulin resistance or beta-cell failure and be complementary when added to insulin-dependent treatments [26,28–33]. Additionally, dapagliflozin significantly reduces body weight and blood pressure across the spectrum of T2DM disease, with maintenance of these benefits over time [15,26,28,33–35]; and it may also improve high density lipoprotein (HDL) cholesterol and triacylglycerol [35]. The adverse effects commonly reported are urinary tract infection (UTI) and genital infection with an overall frequency slightly more than that reported with placebo, which are mainly mild to moderate in severity [28,36–37]. Overall, dapagliflozin has a good tolerability and safety profile with a low propensity to incur hypoglycaemia [15,26,28,33–34]. Dapagliflozin as a monotherapy in treatment-naïve patients with T2DM has been proved to improve glycaemic control and reduce weight without increasing hypoglycaemia [29,38–43]. Specifically, dapagliflozin 10 mg monotherapy demonstrated non-inferiority to metformin monotherapy in lowering HbA1c, but significantly better in reducing fasting plasma glucose, body weight and blood pressure [38,40]. Dapagliflozin may offer an alternative initial medication for treatment-naive patients where diet and exercise alone do not provide adequate glyceamic control or other non-insulin anti-diabetic agents are considered inappropriate [28–29,38,44].

Acarbose is a classical alpha-glucosidase inhibitor used by many Chinese patients with T2DM (35.9%) [45], which is reported to have similar glucose lowering effects to metformin but with higher treatment costs [46–48]. It is recommended as an alternative medication for first-line therapy in China [1]. Therefore, we aimed to examine the cost-effectiveness of dapagliflozin compared with acarbose as a monotherapy for patients with T2DM in China.

However, from previous literature reviews, it was found that there is no direct head-to-head comparison study of dapagliflozin monotherapy versus acarbose monotherapy; while it is more common for them to compare with placebo. Therefore, the objective of this study is to conduct a long-term cost-effectiveness analysis for dapagliflozin versus acarbose as a monotherapy in treatment-naïve patients with type 2 diabetes mellitus (T2DM) in China by identifying efficacy data using meta-analysis and indirect treatment comparison methods by using placebo as a common comparator. This study takes the perspective of health care payers.

## Methods

### Cost-effectiveness model

A previously published and validated economic model, Cardiff Diabetes Model, was used in this study [49–52]. It is a patient level fixed-time increment, Monte Carlo micro simulation model designed to evaluate the long-term cost-effectiveness of comparable diabetes treatments (a “treatment” arm versus a “control” arm). The model utilizes United Kingdom Prospective Diabetes Study 68 (UKPDS 68) risk equations to simulate disease progression and predict the incidences of diabetes-related vascular events (microvascular and macrovascular events), hypoglycaemia and mortality [53]. The model estimates costs and quality adjusted life years (QALYs) associated with each arm, and cost-effectiveness is assessed in terms of the incremental cost per QALY gained (i.e., incremental cost-effectiveness ratio, ICER). We simulated a cohort of 1000 patients with T2DM over a period of 40 years. Patients discontinued the simulation only when death occurred or time horizon was reached. The costs and benefits were discounted at an annual rate of 3% based on the World Health Organization (WHO) guideline [54].

### Literature review

The purpose of the literature review was to obtain information about the disease and collect model-required data for patient profiles, clinical effects of drugs, costs and utilities associated with diabetes-related events.

PubMed, Web of Knowledge (including Web of Science, MEDLINE, BIOSIS Citation Index, Derwent Innovations Index), ScienceDirect and OVID were systematically searched for eligible studies to obtain patients profiles and clinical effects of drugs. Search terms included *dapagliflozin* or *acarbose* in combination with *placebo* and *type 2 diabetes mellitus*. The search period was between 1990.01.01 and 2015.12.31 (Detailed search strategies are provided in S1 Table).

The references and citations of the included studies and relevant reviews were manually examined to identify additional studies. Search results were independently reviewed and screened by two reviewers who then independently extracted the data and evaluated the quality of included studies. Any disagreements between the reviewers were resolved by a discussion, or resolved with consultation of a third reviewer.

Inclusion and exclusion criteria were predefined. Only head-to-head randomized clinical trials (12 weeks or longer) comparing clinical efficacy of dapagliflozin (or acarbose) monotherapy versus placebo monotherapy in patients with T2DM (18 years or older) whose estimated glomerular filtration rate (eGFR) was over 60 mL/min/1.73 m^2^ were included. Because there is a lack of studies in monotherapy of dapagliflozin versus placebo, especially in Chinese patients, we targeted an Asian population to get patient profile and clinical data (Detailed selection criteria are provided in S1 Appendix).

**Fig 1:**
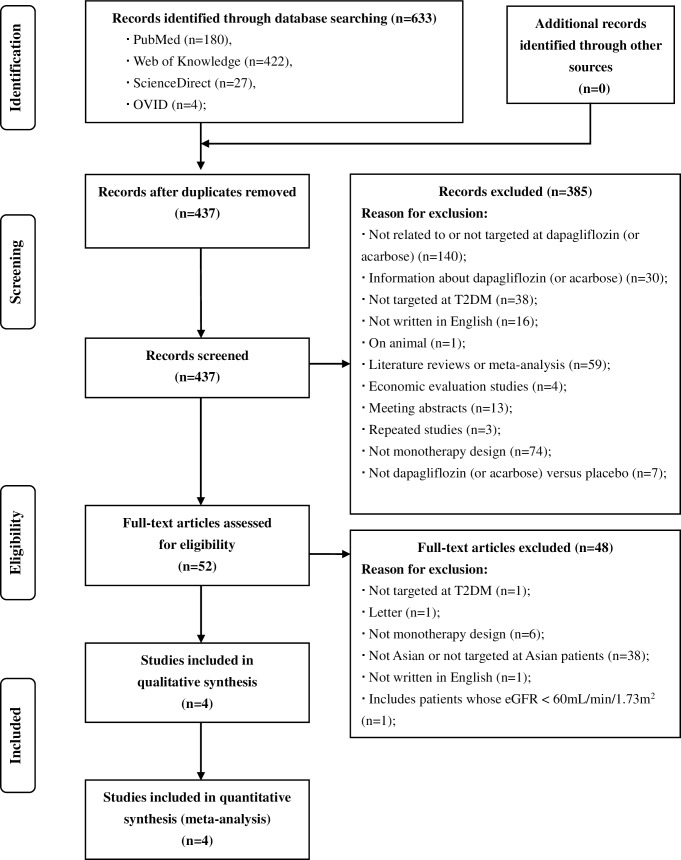
Flow diagram of literature review. A detailed flow diagram that depicts search and selection processes.

A total of 633 potentially relevant records were identified through database searching. 437 records were retrieved for initial screening after duplicates were removed. Title and abstract screening resulted in 52 papers for detailed review. After examination of full-text articles, four eligible studies were finally included in the meta-analysis (two on dapagliflozin versus placebo, and two on acarbose versus placebo) [41–42,55–56] (**Fig 1**).

A standardized extraction form was developed to record the study characteristics, interventions and results. The quality of included studies was assessed using the Cochrane Handbook for Systematic Reviews of Interventions (version 5.1) [57–59] (Characteristics and quality of included studies are provided in S2 and S3 Tables).

### Meta analysis

Six independent meta-analyses were performed for dapagliflozin versus placebo (or acarbose versus placebo) to estimate the combined clinical effects of each interest. Clinical effects included changes of HbA1c, SBP and body weight after treatment (i.e., meta-analysis of dapagliflozin versus placebo on HbA1c, meta-analysis of acarbose versus placebo on HbA1c, meta-analysis of dapagliflozin versus placebo on SBP, meta-analysis of acarbose versus placebo on SBP, meta-analysis of dapagliflozin versus placebo on body weight, meta-analysis of acarbose versus placebo on body weight). As the data were continuous variables, we used weighted mean difference (WMD) and 95% confidence intervals (CI) to describe them. Meta-analyses were carried out by STATA version 11.0. Heterogeneity is quantified by statistic I^2^. A fixed-effect model was used when no significant heterogeneity is detected among studies (P>0.10, I^2^ ≤50%); otherwise, a random-effect model was used. The summary results were reported in **Table 1** (Detailed results of meta-analysis are provided in S1–S6 Figs).

**Table 1:** Summary results of meta-analysis and indirect treatment comparison.

Variable ^a^	Comparison Type	Group	Results [Mean difference (95%CI)]	*P* values
**HbA1c, %**				
	Meta-analysis	Dapagliflozin vs. Placebo	–0.82 [–0.95, –0.68]	< 0.00001
	Meta-analysis	Acarbose vs. Placebo	–0.47 [–0.86, –0.07]	0.02
	ITC	Dapagliflozin vs. Acarbose	–0.35 [–0.767, 0.067]	0.45572
**SBP, mmHg**				
	Meta-analysis	Dapagliflozin vs. Placebo	–4.30 [–6.94, –1.67]	0.001
	Meta-analysis	Acarbose vs. Placebo	–0.17 [–4.51, 4.17]	0.94
	ITC	Dapagliflozin vs. Acarbose	–4.13 [–9.207, 0.947]	--
**Body Weight, kg**				
	Meta-analysis	Dapagliflozin vs. Placebo	–1.91 [–2.31, –1.50]	< 0.00001
	Meta-analysis	Acarbose vs. Placebo	–1.41 [–2.63, –0.18]	0.02
	ITC	Dapagliflozin vs. Acarbose	–0.50 [–1.79, 0.79]	0.79383

HbA1c, glycated hemoglobin; ITC, indirect treatment comparison; SBP, systolic blood pressure.

^**a**^ Variables were taken from data of 4 included placebo-controlled studies [41–42,55–56].

### Indirect treatment comparison

In the absence of a head-to-head study comparing dapagliflozin with acarbose, adjusted indirect treatment comparisons (ITC) for the outcomes of interest were performed by using placebo as a common comparator, to convert the summary estimates from meta-analysis of direct comparisons (i.e., dapagliflozin versus placebo, acarbose versus placebo) into combined WMD and 95% CIs to represent the comparative effect of dapagliflozin versus acarbose. The adjusted ITC uses the method suggested by Bucher et al. in which the comparison of the interventions of interest is adjusted by the results of their direct comparisons with a common intervention control (e.g. placebo), partially using the strength of the randomized clinical trials [60–61]. The Bucher-adjusted method with the ITC calculator developed by Canadian Agency for Drugs and Technologies in Health (CADTH) were used to carry out the ITCs [60,62–63] (**Table 1**).

### Hospital survey

Hospital surveys were conducted in one secondary hospital and one tertiary hospital in eastern China, to collect the real-world cost data for treating model-required diabetes-related complications and use as an alternative to the costs from the literature review. Based on the perspective of health care payers, direct medical costs of diabetes-related complications incurred between 2010 and 2014 were anonymously collected from the Hospital Information System (HIS). We did not contact with any patients, and also did not collected any personal/privacy information about the patients from the HIS. The data were synthesized and converted to form a real-world cost profile (2015 Chinese value) which was tested in the univariate sensitivity analysis.

## Model Inputs

### Patient profile and treatment strategy

The patient cohort was initialized with a set of baseline demographics (e.g., age, percentage of females, duration of diabetes, height and proportion of smokers) and risk factors (e.g., HbA1c, total cholesterol, HDL cholesterol, SBP and body weight). We synthesized the characteristics of patients in the four included placebo-controlled RCTs by using meta-analysis to form a general profile for the model. For data that were not available (e.g. height, proportion of smokers), we used data from national observational studies of patients with T2DM in China [16,64] (**Table 2**).

**Table 2:** Demographics and risk factors.

Variable ^a^	Mean	Standard error
**Baseline Demographics**		
Current Age, y	52.51	0.66
Proportion female, value: 0–1	0.36	0.03
Duration of diabetes, y	2.72	0.22
Height, m	1.65	0
Proportion Afro-Caribbean, value: 0–1	0	0
Proportion Indian, value: 0–1	0.04	0.01
Proportion smokers, value: 0–1	0.183	0.005
**Modifiable Risk Factors**		
HbA1c, %	8.27	0.06
Total-cholesterol, mmol/L	4.96	0.06
HDL cholesterol, mmol/L	1.16	0.02
SBP, mmHg	125.29	1.14
Body weight, kg	67.06	0.68

BMI, body mass index; HbA1c, glycated hemoglobin; HDL, high-density lipoprotein; SBP, systolic blood pressure.

^**a**^ Most variables were taken from pooled data of 4 included placebo-controlled studies [41–42,55–56]. For unavailable data, they were obtained from published observational studies.

Patients entered the model on either dapagliflozin alone (treatment arm, 10mg/d) or acarbose alone (control arm, 300mg/d). In the case of inadequate glycaemic control (HbA1c > 8%), therapy escalation occurred: patients in both arms were switched to metformin + sulfonylureas regimen for rescue, and then switched to insulin regimen if above therapies failed to achieve glycaemic control.

### Clinical effect and adverse effect

The results of the ITC showed that dapagliflozin reduced HbA1c levels (-0.35%), SBP (-4.13 mmHg) and body weight (-0.50 kg) more than acarbose, but the differences were not statistically significant. Considering the data required by the model design which uses the absolute change from baseline rather than relative change, the synthesized efficacy of dapagliflozin from the meta-analysis of two included placebo-controlled RCTs on dapagliflozin was directly used as the model input; and the efficacy of acarbose was calculated based on the efficacy of dapagliflozin and the ITC results. The incidences of hypoglycaemia and other adverse events (e.g., urinary tract infection, genital infection, gastrointestinal adverse effect) induced by both drugs and the rates of therapy discontinuation of both drugs were synthesized from the meta-analysis of the included placebo-controlled RCTs. Because incidences of hypoglycaemia were not reported in the included RCTs on acarbose, we used the rates reported by an open-label, non-inferiority randomised trial of acarbose in Chinese patients newly diagnosed with T2DM as a reference [47] (**Table 3**).

**Table 3:** Clinical input variables.

	Dapagliflozin		Acarbose	
Variable ^a^	Mean	SE	Mean	SE
HbA1c reduction, %	–0.79	0.05	–0.44	0.22
SBP, mmHg	–3.28	0.93	0.85	2.75
Body weight change, kg	–2.05	0.15	–1.55	0.67
Probability symptomatic hypoglycaemia	0.011	0.008 ^**b**^	0.010	0.005 ^**b**^
Probability severe hypoglycaemia	0	0	0	0
Urinary tract infection	0.049	0.016 ^**b**^	--	--
Genital infection	0.032	0.013 ^**b**^	--	--
Gastrointestinal adverse effect	--	--	0.582	0.054 ^**b**^
Therapy discontinuation	0.086	0.021	0.146	0.039

HbA1c, glycated hemoglobin; SE, standard error; SBP, systolic blood pressure.

^**a**^ Most variables were taken from pooled data of 4 included placebo-controlled studies [41–42,55–56]. Hypoglycaemia induced by acarbose was from a randomised trial [47].

^**b**^ Calculated as √ rate (1–rate)/numbers of subjects.

### Costs

Costs associated with diabetes-related vascular events, adverse events, body mass index (BMI) changes and drug acquisitions were included in this study. From the perspective of health care payers, only direct medical costs were considered, and all costs were converted to 2015 (Chinese Yuan, ¥) using the Chinese Consumer Price Index (CPI) [65]. One US dollar was equal to 6.227 Yuan in 2015 [66].

Costs for vascular events included fatal and non-fatal costs (applied in the year when the event occurred) and maintenance costs (applied in subsequent years if patients survived or simulation horizon was not reached); and the costs were primarily based on a published Chinese study [67]. As the costs of ulcers were not available, they were derived by synthesizing data from other published studies [68–69] (**Table 4**).

**Table 4:** Annual direct medical costs for diabetes-related events and adverse events (2015 Chinese value).

	Fatal		Nonfatal		Maintenance	
Event ^a^	Mean	SE	Mean	SE	Mean	SE
Ischemic heart disease	--	0	38660.37	0	6901.83	0
Myocardial infarction	46092.75	0	46092.75	0	10588.10	0
Congestive heart failure	15328.57	0	15328.57	0	9317.53	0
Stroke	13922.20	0	17964.09	0	8089.54	0
Blind	--	--	11930.02	0	9207.04	0
End-stage renal disease	--	--	113521.66	0	91084.11	0
Amputation	18055.01	0	18055.01	0	14391.76	0
Ulcer	0	0	14190.51	443.2	4994.42	0
Severe hypoglycemia	--	--	3787.91	0	--	--
Urinary tract infection ^**b**^	--	--	201.7	0	--	--
Genital infection ^**b**^	--	--	201.7	0	--	--
Gastrointestinal adverse	--	--	0	0	--	--

SE, standard error.

^**a**^ Costs for vascular events were primarily based on a published Chinese study [67]. Costs of ulcer were derived by synthesizing data from other published studies [68–69]. Costs for severe hypoglycemia [70], urinary tract infection and genital infection [71] were taken from published studies. Cost for gastrointestinal adverse was assumed to be 0.

^**b**^ For urinary tract infection and genital infection, only drug costs were considered.

Hypoglycaemic events were assumed to be either mild, moderate or severe, where severe was defined as an episode that requires medical assistance [72–73]. The costs for severe hypoglycaemia and other adverse events such as urinary tract infection and genital infection were extracted from published studies [70–71]; while for gastrointestinal adverse events, it was assumed to be zero (**Table 4**). BMI-related costs relating to increased prescribing costs per BMI unit were estimated from an observational study in China [74] (**Table 5**).

**Table 5:** Body mass index (BMI) -related costs ^a^ (2015 Chinese value).

BMI	Annual Cost	BMI	Annual Cost	BMI	Annual Cost
20	0	27	8484.9	34	24609.4
21	0	28	10788.4	35	26912.9
22	0	29	13091.9	36	29216.4
23	0	30	15395.4	37	31519.9
24	1574.4	31	17698.9	38	33823.4
25	3877.9	32	20002.4	39	36126.9
26	6181.4	33	22305.9	40+	38430.4

^**a**^ Variables were estimated from Guo et al [74]. Assumptions: The starting point BMI = 25, cost per month = ¥246.8, the slope (cost per month/ BMI) = ¥146.6 in 2007. For BMI≤23 the cost was set to zero.

The drug acquisition cost for acarbose is estimated according to the highest retail price from the official drug price list of the Price Bureau in eastern China [75]. As dapagliflozin is unavailable in the Chinese medical market, we set an assumption on its highest retail price based on Hong Kong price which is obtained from AstraZeneca in March 2016. As the price for a package of Farxiga (10mg*28 tablet) varies in different areas of Hong Kong ($530 in Shaukiwan, $530 in Kowloon City, and $550 in Central), we assumed a middle price of $540 as the highest retail price in our base case analysis, and used the price of $530 and $550 as an alternative price profile which were tested in the univariate sensitivity analysis. We used the current exchange rate and Chinese CPI to convert the prices to 2015 Chinese values [65,76]. Daily drug dosages were obtained from the included studies (dapagliflozin: 10mg/d; acarbose: 300mg/d) [41–42,55–56] (**Table 6**).

**Table 6:** Annual treatment costs for different drugs (2015 Chinese value).

Drug (Brand)	Manufacturer	Specification	Highest Retail Price, ¥	Daily Dose, mg/d	Daily drug cost, ¥	Annual drug cost, ¥
Dapagliflozin (Farxiga)	AstraZeneca	10mg*28 tablet	446.37 ^**a**^	10	15.94	5826.72
Acarbose (Glucobay)	Bayer Health Care	50mg*30 tablets	76.77 ^**b**^	300	15.35	5611.89
Metformin (Glucophage)	Bristol-Myers Squibb	500mg*20 tablets	30.21 ^**c**^	1500	4.53	1656.26
Sulfonylureas (Glimepiride)	Sanofi	2mg* 15 tablets	80.60 ^**d**^	4	10.75	3927.91

^**a**^ Price of dapagliflozin was assumed based on Hong Kong price which was obtained from AstraZeneca in March, 2016. Price in 2016 in Hong Kong is $540, convert to Chinese value and inflation to 2015 is ¥446.37.

^**b**^ Official drug price list of Price Bureau for acarbose in eastern China [75]. Price in 2013 is ¥74.2, inflation to 2015 is ¥76.77.

^**c**^ Official drug price list of Price Bureau for metformin in eastern China [75]. Price in 2013 is ¥29.2, inflation to 2015 is ¥30.21.

^**d**^ Annual cost of glimepiride was used as the cost of sulfonylureas. Official drug price list of Price Bureau for glimepiride in eastern China [75]. Price in 2013 is ¥77.90, inflation to 2015 is ¥80.60.

### Utilities

In the absence of country-specific utilities for diabetes-related events in China, most of the utilities were adopted from the UKPDS 62 study [77]. For those unavailable in UKPDS 62 study, including end-stage renal disease (ESRD) and blindness [78], hypoglycaemia [79], per unit change in BMI [80], gastrointestinal adverse events [81] and urinary tract infection (UTI) [82], values were obtained from other published studies. Given the lack of utility data available for genital infections, it was assumed to be equivalent to that of UTI (**Table 7**).

**Table 7:** Utility decrements.

Event ^a^	Utility Decrement
Ischemic heart disease	0.090
Myocardial infarction	0.055
Congestive heart failure	0.108
Stroke	0.164
Blind	0.074
End-stage renal disease	0.263
Amputation	0.280
Ulcer	0.059
Symptomatic hypoglycaemia	0.0142
Severe hypoglycaemia	0.047
Urinary tract infection	0.003
Genital infection	0.003
Gastrointestinal adverse	0.040
Per unit decrease in BMI	0.0171
Per unit increase in BMI	0.0472

BMI, body mass index.

^**a**^ Utility decrements for most events were adopted from the UKPDS 62 study [77], except for end-stage renal disease and blindness [78], hypoglycaemia [79], BMI-related changes [80], gastrointestinal adverse events [81] and urinary tract infection [82]. Utility for genital infections was assumed to be equivalent to urinary tract infection.

### Sensitivity analyses

Univariate sensitivity analysis and probabilistic sensitivity analysis (PSA) were conducted to assess the impact of uncertainty around model inputs including baseline demographics, costs and utilities assigned to diabetes-related events and BMI changes, etc.

In the univariate sensitivity analysis, a variety of different model inputs was used to test the impact of data variations on the conclusion, such as alternative incidents and utility decrements of diabetes-related events, alternative discount rate and therapy escalation threshold. As the drug price of dapagliflozin was assumed based on Hong Kong price in the base case analysis which may result in uncertainty, three alternative drug treatment costs of dapagliflozin were tested. As BMI-related costs had a large impact on the model, the BMI-costs set at 50%, 25% and 0% of the base case input were also tested.

In the PSA, treatment-related HbA1c effects, weight changes and SBP changes were sampled from a normal distribution; the utility decrements were modeled with a beta distribution; and the costs followed a gamma distribution. The range of values was expressed by the 95% confidence intervals for each parameter. A scatter plot of the ICER and a cost-effectiveness acceptability curve (CEAC) were generated. All of the sensitivity analyses were performed for 1000 patients.

## Results

### Predicted health events

**Fig 2:**
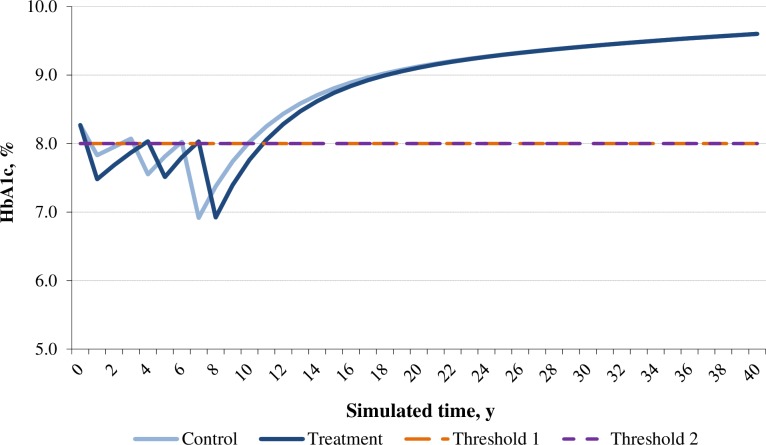
Simulated progression of HbA1c in the treatment (dapagliflozin) and control (acarbose) arms over the modeled time horizon.

**Fig 3:**
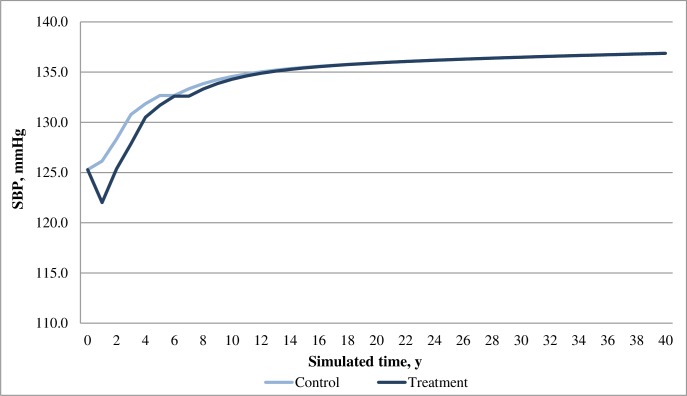
Simulated progression of SBP in the treatment (dapagliflozin) and control (acarbose) arms over the modeled time horizon.

**Fig 4:**
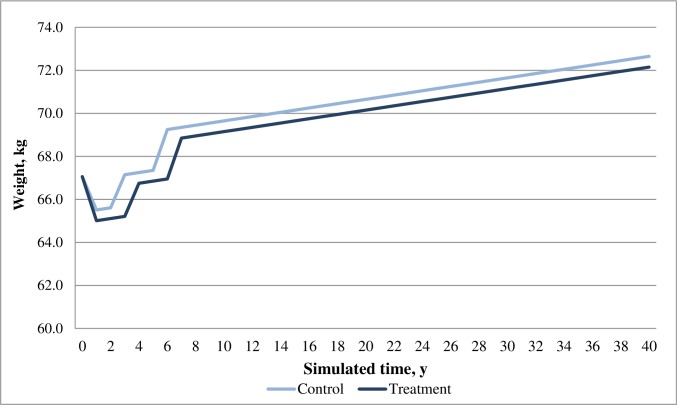
Simulated progression of body weight in the treatment (dapagliflozin) and control (acarbose) arms over the modeled time horizon.

In the base case analysis, both dapagliflozin and acarbose arms showed positive effects in reducing HbA1c and body weight for patients over time; while the dapagliflozin arm was associated with greater reduction in both HbA1c and weight compared with the acarbose arm. Moreover, dapagliflozin was associated with a decrease in SBP whilst acarbose was associated with an increase in SBP (**Figs 2–4**). Overall, the model predicted lower incidences of both macrovascular and microvascular events in the dapagliflozin arm compared with the acarbose arm. Correspondingly, the dapagliflozin arm was also associated with fewer predicted deaths caused by vascular events. Furthermore, patients in the dapagliflozin arm experienced fewer symptomatic and severe hypoglycaemia events than that in the acarbose arm (**Table 8**).

**Table 8:** Base case results for dapagliflozin arm versus acarbose arm.

Total Events Predicted	Acarbose		Dapagliflozin		Difference	Total Costs, ^a^ ¥	Acarbose	Dapagliflozin
Macrovascular	Non-Fatal	Fatal	Non-Fatal	Fatal		Macrovascular		
Ischemic heart disease	123.85	0	122.34	0	–1.51	Ischemic heart disease	8,874,972	8,726,979
Myocardial infarction	132.04	167.93	131.13	166.40	–2.43	Myocardial infarction	17,815,873	17,609,167
Congestive heart failure	54.31	5.71	54.07	5.70	–0.25	Congestive heart failure	2,892,398	2,873,406
Stroke	63.21	17.47	62.65	17.13	–0.89	Stroke	3,743,474	3,667,441
Microvascular						Microvascular		
Blindness	63.80	0	63.12	0	–0.68	Blindness	4,286,342	4,242,735
Nephropathy	21.73	2.24	21.50	2.28	–0.18	Nephropathy	8,446,813	8,256,814
Amputation	36.83	3.87	36.00	3.78	–0.92	Amputation	2,634,415	2,550,328
Fatal						Hypoglycaemia	973,750	899,395
Macrovascular	--	191.11	--	189.23	–1.89	Adverse Events	10,001	66,175
Microvascular	--	6.11	--	6.06	–0.05	Treatment	65,845,462	68,371,894
						BMI Costs	53,925,955	43,745,715
						Total	169,449,456	161,010,049
Cost-Effectiveness (Per Patient)	Acarbose		Dapagliflozin		Difference	Hypoglycaemia ^**b**^	Acarbose	Dapagliflozin
Discounted Cost	169,449.46		161,010.05		–8,439	Symptomatic	11,391	10,786
Discounted QALYs	12.99		13.24		0.25	Severe	386	364
Discounted LYs	15.82		15.86		0.04			
Cost per QALY			Dominant		–33,786			
Cost per LY			Dominant		–229,566			

BMI, body mass index; LY, life year; QALY, quality-adjusted life-year.

^**a**^ Treatment costs include metformin + sulfonylureas therapy and insulin therapy drug costs. Analysis based on 1000 patients.

^**b**^ Hypoglycemia in both the treatment and the control group includes hypoglycemia events generated by metformin + sulfonylureas therapy and insulin therapy.

### Predicted costs

Consistent with the differences in cases of macrovascular and microvascular events, costs for these events were all lower in the dapagliflozin arm compared with the acarbose arm. Although the dapagliflozin arm was associated with higher drug costs and treatment-induced adverse event costs compared with acarbose, this tiny disadvantage could not greatly affect the total costs because both vascular event related costs and BMI-related costs were lower with the dapagliflozin arm compared with the acarbose arm. The dapagliflozin arm was associated with lower total costs of long-term diabetes treatments compared with the acarbose arm (**Table 8**).

### Incremental cost-effectiveness ratio (ICER)

For an individual patient, the discounted costs accumulated over 40 years in the dapagliflozin arm and in the acarbose arm were ¥161,010.05and ¥169,449.46, respectively; and the discounted QALYs were 13.24 and 12.99, respectively. The dapagliflozin arm was associated with a mean incremental benefit of 0.25 QALYs and with a lower cost of ¥8,439 compared with the acarbose arm. This resulted in a cost saving of ¥33,786 per QALY gained with dapagliflozin compared with acarbose. The results showed that dapagliflozin dominates acarbose with lower costs and higher QALY gains per patient over a 40 year time horizon (**Table 8**).

### Parameters influencing the incremental cost-effectiveness ratio

**Fig 5:**
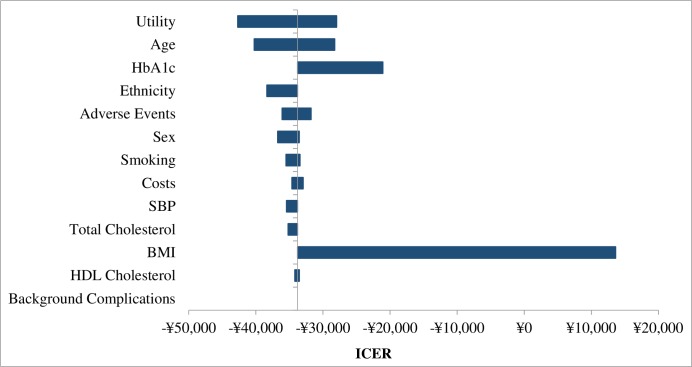
Tornado diagram of the univariate sensitivity analysis.

Results of the sensitivity analyses demonstrated the robustness of the base case results produced by the model which calculated that dapagliflozin treatment was cost saving and generated more QALY benefit compared with acarbose treatment (**Table 9**). **Fig 5** presents univariate sensitivity analyses as a tornado diagram. BMI was identified as the most influential factor affecting the ICER result. Dapagliflozin remained dominant both when baseline age changed from 40–70 years, and when baseline HbA1c was changed to the upper (+25%) and lower (-25%) limit percentage (ICER results available upon request).

**Table 9:** Sensitivity analyses results for dapagliflozin arm versus acarbose arm (results per patient).

Sensitivity Analysis ^a^	Difference in Cost, ¥	Difference in QALY	ICER, ¥
**Univariate sensitivity analysis**			
Utility weight 0.014 per unit BMI decrease and -0.014 per unit BMI increase in all years	–8,439	0.16	–53,618
Utility decrement per unit BMI gain halved in all years	–8,439	0.19	–45,149
Utility decrement per unit BMI gain reduced by 75% in all years	–8,439	0.16	–54,275
BMI-related costs halved	–3,349	0.25	–13,408
BMI-related costs reduced by 75%	–804	0.25	–3,220
BMI-related costs = 0	1,741	0.25	6,969
All hypoglycemia of both drugs be severe events	–8,389	0.25	–33,642
All hypoglycemia of both drugs = 0	–8,439	0.25	–33,760
Utility decrement of symptomatic and severe hypoglycemia halved	–8,439	0.25	–34,379
Urinary tract and genital infection events rates of dapagliflozin = 0	–8,496	0.25	–33,900
Utility decrement of urinary tract and genital infection events halved	–8,439	0.25	–33,730
Gastrointestinal adverse events rate of acarbose = 0	–8,439	0.19	–43,665
Utility decrement of gastrointestinal adverse events halved	–8,439	0.22	–38,095
HbA1c threshold value for therapy switch 7.0%	–4,481	0.20	–22,426
Discount rate for costs and benefits 5%	–7,116	0.21	–33,356
Annual treatment cost of dapagliflozin equal to acarbose	–9,184	0.25	–36,768
Annual treatment cost of dapagliflozin (¥5718.77)	–8,814	0.25	–35,284
Annual treatment cost of dapagliflozin (¥5934.68)	–8,065	0.25	–32,287
Alternative costs of diabetes-related complications	–8,436	0.25	–33,772
Costs of diabetes-related complications halved	–8,056	0.25	–32,250
Utility decrement of diabetes-related complications halved in all years	–8,439	0.25	–34,154
**Probabilistic sensitivity analysis**	**–6,837**	**0.26**	**–26,109**

BMI, body mass index; HbA1c, glycated hemoglobin; QALY, quality-adjusted life-year; ICER, incremental cost-effectiveness ratio.

^**a**^ Analysis for 1000 patients. Everything else is as described for the base case analysis.

In the scenario where an alternative utility profile associated with body weight changes was used, in which the utility change value related to either gain or loss per unit BMI was set to be 0.014 [83]; the incremental QALYs decreased from 0.25 to 0.16 with a reported ICER of–¥53,618/QALY for dapagliflozin versus acarbose, which provided a 59% absolute increase in the ICER result compared with the base case (–¥33,786/QALY). In the scenarios where the utility decrement per unit BMI gain was halved or reduced by 75%, the incremental QALYs decreased from 0.25 to 0.19 (or 0.16), while the resulting cost savings per QALY gained with dapagliflozin still remained high at–¥45,149/QALY or–¥54,275/QALY (i.e., absolute increases of 34% or 61%). Dapagliflozin dominated acarbose, increasing QALYs and reducing costs. A decrease in BMI-related costs would have a negative effect on the ICER result, but the results still favored dapagliflozin. Either when BMI-related costs were halved or reduced by 75%, dapagliflozin remained dominant with reported ICERs of–¥13,408/QALY and–¥3,220/QALY (i.e., absolute decreases of 60% and 90% versus base case), respectively. Only when BMI-related costs were excluded from the model did the dapagliflozin arm cost more than acarbose arm with a reported ICER of ¥6,969/QALY, which was within the acceptable range of 2014 GDP per capita of China (¥46,629) [84] (**Table 9**).

Although treatment-induced hypoglycemia, UTI and genital infection were associated with utility decrements and, for severe hypoglycemic events, a related cost, the adjustment of event rates and utility decrement associated with them in alternative scenarios led to only negligible effects on the magnitude of cost savings by dapagliflozin. Conversely, gastrointestinal adverse events induced by acarbose had more implication on the ICER. Either when gastrointestinal adverse events were set to be 0 or when utility decrement of gastrointestinal adverse events was halved, the incremental QALYs decreased from 0.25 to 0.19 (or 0.22), with a 29% (or 13%) absolute increase of ICER (–¥43,665/QALY or–¥38,095/QALY) versus that of base case (–¥33,786/QALY). The impacts of adjusting the therapy escalation threshold (decreased to HbA1c = 7%) was also investigated, the dapagliflozin arm remained cost effective with a 34% absolute decrease of ICER (–¥22,426/QALY) versus the base case. The ICER was not sensitive to changes in the annual treatment cost of dapagliflozin, discount rate for costs and benefits, costs of diabetes-related complications, utility decrement of diabetes-related complications (**Table 9**).

**Fig 6:**
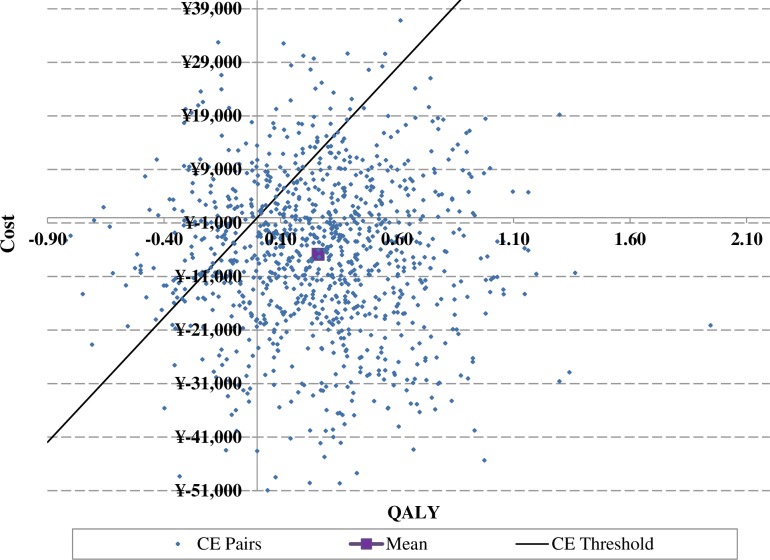
Scatter plot of incremental cost-effectiveness ratios for the treatment (dapagliflozin) arm versus control (acarbose) arm with a CE threshold value of ¥46,629 (GDP per capita in China in 2014).

**Fig 7:**
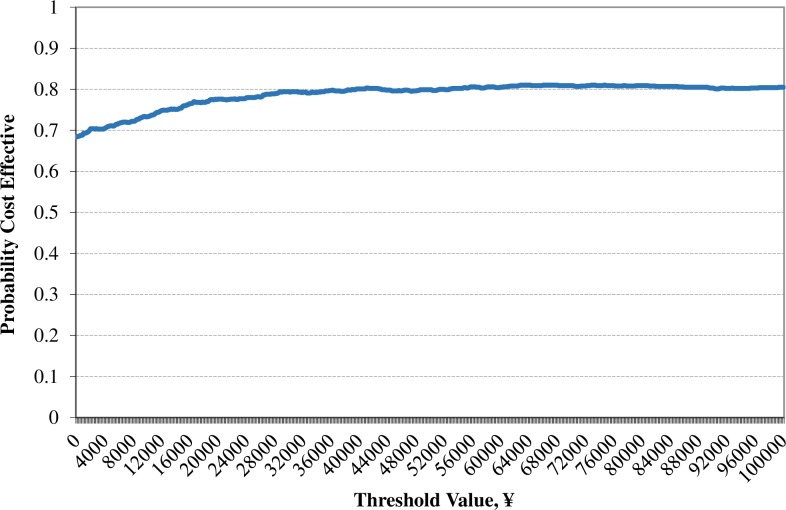
Cost effectiveness acceptability curve for the treatment (dapagliflozin) arm versus control (acarbose) arm.

In the PSA, dapagliflozin arm generated a mean incremental QALY gained of 0.26 versus the acarbose arm similar to that of the base case; whilst the mean incremental cost was–¥6,837, which was lower than that of base case (–¥8,439). As a result, the ICER of–¥26,109/QALY for dapagliflozin versus acarbose in the PSA was lower than that of base case (–¥33,786/QALY) (**Table 9**). Most simulations were located in the southeast quadrant of the ICER scatter plot figure, which means dapagliflozin arm gained more benefits for a lower cost than the acarbose arm. The dapagliflozin arm was cost-effective in 79.8% of the simulations using a cost-effective threshold value of ¥46,629 (GDP per capita in China in 2014) [84] (**Figs 6 and 7**).

## Discussion

This study is the first pharmacoeconomic analysis conducted using the Cardiff Diabetes Model to assess the long-term cost-effectiveness of dapagliflozin versus acarbose as a monotherapy for T2DM from the perspective of health care payers in China. The efficacy data were abstracted by combing the methods of meta-analysis and ITC of RTCs (dapagliflozin versus placebo and acarbose versus placebo) by using placebo as a common comparator. The base case results demonstrated that dapagliflozin monotherapy was a dominant therapy (with higher total QALYs gained but lower total costs) compared with acarbose monotherapy, which may offer an alternative initial medication for treatment-naive patients where diet and exercise alone do not provide adequate glyceamic control or other non-insulin anti-diabetic agents are considered inappropriate. The cost-effectiveness results were robust to changes in relevant model input parameters in a series of sensitivity analyses.

Both hypertension and being overweight (or obesity) are common comorbidities of diabetes with weight gain being a common adverse effect of some diabetes treatments [1,9]. Hypertension and excess weight have negative effects on patients’ quality of life, and increase the risks for cardiovascular morbidity and mortality, and continually increase economic burden [16,22,85–90]. It was reported that tight glucose control and blood pressure control achieved clinically important reductions in the risk of diabetes-related deaths and complications [6–8]; while even modest weight losses of 5 to <10% were associated with significant improvements in cardiovascular disease risk factors (e.g., HbA1c, blood pressure and triglycerides) [91]. In this study, it was found that dapagliflozin was numerically associated with greater reduction in HbA1c, SBP and body weight compared with acarbose; which would account for the lower incidences of cardiovascular events and relevant mortality in the dapagliflozin arm, and further account for the better benefits and lower total costs achieved by dapagliflozin arm for patients over 40 years treatment compared with acarbose. Sensitivity analysis partly confirmed this finding, as adjustments of baseline HbA1c and BMI, and changes on both utility and cost values associated with a unit change in BMI in sensitivity analysis had great implications for the ICER results.

On the other hand, treatment-induced adverse events like hypoglycaemia and gastrointestinal adverse events are also common in diabetes treatment [1,9], which may negatively impact patients’ quality of life and treatment adherence [20,79]. In this study, because patients in both dapagliflozin and acarbose treatments only experienced few symptomatic hypoglycaemia events which were not significantly different in both arms, hypoglycaemia might not significantly affect the ICER results. Since dapagliflozin was associated with low incidences of adverse events like UTI and genital infections, these events might also not significant impact the ICER results. Conversely, the comparative more commonly observed gastrointestinal adverse events caused by acarbose had more impact on the ICER. Sensitivity analyses on these events also confirmed the findings.

Poor medication adherence is a common and costly public health challenge, and it frequently occurs in the medications of chronic diseases like diabetes [92]. None or suboptimal adherence is related to poorer treatment outcomes, progression of disease symptoms and complications, increased risks of hospitalizations and mortality, and higher costs [23,92–95]. Efficacy and tolerability are no longer the only criterion to assess a drug, factors such as ease of administration, convenient dosing frequency, beneficial weight control profile, low risks of hypoglycaemia or other adverse events which were associated with better adherence, are also essential [21,96–98]. Acarbose is an oral drug administered to patients 3 times daily and should be chewed with the first mouthful of food, or swallowed whole with a little liquid directly before the meal; while dapagliflozin is administered less frequently and more easily (taken orally once daily by swallowing the whole tablet at any time of day with or without food) [44,99]. Therefore, patients with dapagliflozin may have better medication adherence and fewer therapy discontinuations as compared with acarbose in some extent. In this study, according to the reports of included RCTs, therapy discontinuation rates were 8.6% and 14.6% in dapagliflozin and acarbose treatment, respectively, with reasons such as intolerance to therapy or adverse events, lost to follow-up, poor glycaemic control, poor medication adherence, or no longer met study criteria [41–43,55–56]. Potential better therapy adherence may be another advantage of dapagliflozin.

Treatment cost is also a public concern in long-term care for T2DM. Since the official cost of dapagliflozin is not available in China, we assumed several highest retail prices for dapagliflozin both in the base case analysis and in the univariate sensitivity analysis based on the Hong Kong price. The results showed that whatever its annual cost was equal to or higher than that of acarbose, dapagliflozin arm dominates acarbose arm with lower total costs and higher QALY gains. Favorable treatment cost seems to be an additional advantage of dapagliflozin for long-term diabetes care.

Regarding the poor management, suboptimal medication adherence, high relevant deaths and great economic burden of diabetes in China; dapagliflozin as an effective drug on controlling blood glucose, blood pressure and body weight, with well-tolerated profile, low risks of hypoglycemia and ease of administration. Its use is anticipated to provide a cost-effective alternative initial medication for patients with T2DM, to consolidate the alternative medication for first-line therapy in China.

Although there is a lack of similar studies of dapagliflozin in monotherapy for T2DM conducted to confirm these findings, several cost-effectiveness studies of dapagliflozin as an add-on therapy to metformin (or insulin) exist. When added to metformin, dapagliflozin was reported to be a cost-effective treatment option compared with dipeptidyl peptidase-4 inhibitors, sulphonylureas or thiazolidinediones in patients inadequately controlled on metformin alone [25,100–102]. Moreover, dapagliflozin in combination with insulin was also estimated to be a cost-effective treatment for patients who failed to get adequate glycaemic control with insulin alone [103]. These studies confirmed the beneficial profile of dapagliflozin for treating T2DM.

The present study is limited by the absence of head-to-head studies of dapagliflozin versus acarbose. Therefore, we combined the methods of meta-analysis and ITC by using placebo as a common comparator to get an integrated knowledge of the clinical efficacy of dapagliflozin versus acarbose, which might cause uncertainty in the input parameters. As with other Cardiff modeling studies, this study projected long-term outcomes based on clinical input parameters from short-term trials by using UKPDS 68 equations [25,100–102]; thus the results might not accurately reflect outcomes in real world settings in China. Additionally, utility decrements for diabetes-related events were mainly obtained from the UKPDS 62 study and other published foreign studies due to the paucity of country-specific data for specific antidiabetic drugs such as dapagliflozin or acarbose established in China yet. On the other hand, only direct medical costs were included in this study given the research perspective taken, which might neglect the great impacts of diabetes-related events and treatment-induced adverse events (e.g., hypoglycaemia, UTI, genital infection and gastrointestinal adverse effect) on productivity (indirect costs). Finally, total costs of the acarbose arm were underestimated due to lack of costs associated with gastrointestinal events, which had undermined the comparability of the treatment arms to some extent for gastrointestinal adverse event had certain impact on ICER.

## Conclusion

Dapagliflozin is a cost-effective treatment alternative for patients as a monotherapy in treating T2DM from the perspective of health care payers in China, demonstrating a little QALY gain and lower costs compared with acarbose monotherapy. Dapagliflozin may offer a well-tolerated and cost-effective alternative initial medication for treatment-naive patients with T2DM in China; it may address some of the unmet medical needs due to adverse events (e.g., hypoglycemia, weight gain, gastrointestinal adverse event) or inconvenient drug administration in the treatment of T2DM, and continually to reduce the disease burden of T2DM.

## Supporting Information

S1 Checklist PRISMA 2009 Checklist. (DOC)

S1 Appendix Selection Criteria. (PDF)

S1 Fig Meta-analysis of dapagliflozin versus placebo on HbA1c. (PDF)

S2 Fig Meta-analysis of acarbose versus placebo on HbA1c. (PDF)

S3 Fig Meta-analysis of dapagliflozin versus placebo on SBP. (PDF)

S4 Fig Meta-analysis of acarbose versus placebo on SBP. (PDF)

S5 Fig Meta-analysis of dapagliflozin versus placebo on weight. (PDF)

S6 Fig Meta-analysis of acarbose versus placebo on weight. (PDF)

S1 Table Search strategy. (PDF)

S2 Table Characteristics of the included studies. (PDF)

S3 Table Quality of the included studies. (PDF)

## References

[1] Chinese Diabetes Society. Chinese guideline for Type 2 diabetes prevention (2013). Chinese Journal of Diabetes. 2014; 22: 2–42.

[2] International Diabetes Federation. IDF DIABETES ATLAS—Seventh Edition 2015. http://www.diabetesatlas.org/. Accessed January 21, 2016.

[3] Xu Y, Wang L, He J, Bi Y, Li M, Wang T, et al Prevalence and control of diabetes in Chinese adults. JAMA. 2013; 310: 948–959. 10.1001/jama.2013.168118 24002281

[4] International Diabetes Federation. About diabetes: types of diabetes. Brussels, Belgium. http://www.idf.org/about-diabetes. Accessed January 21, 2016.

[5] UK Prospective Diabetes Study UKPDS Group. Intensive blood-glucose control with sulphonylureas or insulin compared with conventional treatment and risk of complications in patients with type 2 diabetes (UKPDS 33). Lancet. 1998; 352: 837–853. 9742976

[6] UK Prospective Diabetes Study Group. Tight blood pressure control and risk of macrovascular and microvascular complications in type 2 diabetes: UKPDS 38. BMJ. 1998; 317: 703–713. 9732337 PMC28659

[7] Holman RR, Paul SK, Bethel MA, Matthews DR, Neil HA. 10-year follow-up of intensive glucose control in type 2 diabetes. N Engl J Med. 2008; 359: 1577–1589. 10.1056/NEJMoa0806470 18784090

[8] Stratton IM, Adler AI, Neil HA, Matthews DR, Manley SE, Cull CA, et al Association of glycaemia with macrovascular and microvascular complications of type 2 diabetes (UKPDS 35): prospective observational study. BMJ. 2000; 321: 405–412. 10938048 10.1136/bmj.321.7258.405 PMC27454

[9] Inzucchi SE, Bergenstal RM, Buse JB, Diamant M, Ferrannini E, Nauck M, et al Management of hyperglycaemia in type 2 diabetes: a patient-centered approach. Position statement of the American Diabetes Association (ADA) and the European Association for the Study of Diabetes (EASD). Diabetologia. 2012; 55: 1577–1596. 10.1007/s00125-012-2534-0 22526604

[10] Freeman JS. Managing hyperglycemia in patients with type 2 diabetes mellitus: rationale for the use of dipeptidyl peptidase-4 inhibitors in combination with other oral antidiabetic drugs. J Am Osteopath Assoc. 2010; 110: 528–537. 20876838

[11] Wang W, McGreevey WP, Fu C, Zhan S, Luan R, Chen W, et al Type 2 diabetes mellitus in China: a preventable economic burden. Am J Manag Care. 2009; 15: 593–601. 19747024

[12] Tang L, Chen X, Zhao H, Zhao L, Hu S. The financing burden of treatment of diabetes Ⅱ and Its symptom in urban China. Chinese Health Economics. 2003; 22: 21–23.

[13] Lim SS, Vos T, Flaxman AD, Danaei G, Shibuya K, Adair-Rohani H, et al A comparative risk assessment of burden of disease and injury attributable to 67 risk factors and risk factor clusters in 21 regions, 1990–2010: a systematic analysis for the Global Burden of Disease Study 2010. Lancet. 2012; 380: 2224–2260. 10.1016/S0140-6736(12)61766-8 23245609 PMC4156511

[14] World Health Organization. Comparative quantification of health risks: global and regional burden of disease attribution to selected major risk factors. http://www.who.int/healthinfo/global_burden_disease/cra/en/. Accessed January 21, 2016.

[15] Oliva RV, Bakris GL. Blood pressure effects of sodium-glucose co-transport 2 (SGLT2) inhibitors. J Am Soc Hypertens. 2014; 8: 330–339. 10.1016/j.jash.2014.02.003 24631482

[16] Ji L, Hu D, Pan C, Weng J, Huo Y, Ma C, et al Primacy of the 3B approach to control risk factors for cardiovascular disease in type 2 diabetes patients. Am J Med. 2013; 126: 911–925. 10.1016/j.amjmed.2013.02.035 23810406

[17] Centers for Disease Control and Prevention. Prevalence of overweight and obesity among adults with diagnosed diabetes—United States, 1988–1994 and 1999–2002. Morbidity and Mortality Weekly Report. 2004; 53: 1066–1068. 15549021

[18] International Diabetes Federation Guideline Development Group. Global guideline for type 2 diabetes. Diabetes Res Clin Pract. 2014; 104: 1–52. 10.1016/j.diabres.2012.10.001 24508150

[19] American Diabetes Association. Standards of medical care in diabetes—2014. Diabetes Care. 2014; 37 Suppl 1: S14–S80. 24357209 10.2337/dc14-S014

[20] Alvarez GF, Tofe PS, Krishnarajah G, Lyu R, Mavros P, Yin D. Hypoglycaemic symptoms, treatment satisfaction, adherence and their associations with glycaemic goal in patients with type 2 diabetes mellitus: findings from the Real-Life Effectiveness and Care Patterns of Diabetes Management (RECAP-DM) Study. Diabetes Obes Metab. 2008; 10 Suppl 1: 25–32. 18435671 10.1111/j.1463-1326.2008.00882.x

[21] Larkin AT, Hoffman C, Stevens A, Douglas A, Bloomgarden Z. Determinants of adherence to diabetes treatment. J Diabetes. 2015; 7: 864–871. 10.1111/1753-0407.12264 25565088

[22] Pi-Sunyer FX. The Impact of Weight Gain on Motivation, Compliance, and Metabolic Control in Patients with Type 2 Diabetes Mellitus. POSTGRADUATE MEDICINE. 2009; 121: 94–107. 10.3810/pgm.2009.09.2056 19820278 PMC2879281

[23] Sokol MC, McGuigan KA, Verbrugge RR, Epstein RS. Impact of medication adherence on hospitalization risk and healthcare cost. MEDICAL CARE. 2005; 43: 521–530. 15908846 10.1097/01.mlr.0000163641.86870.af

[24] Lundkvist J, Berne C, Bolinder B, Jonsson L. The economic and quality of life impact of hypoglycemia. The European journal of health economics. 2005; 6. 10.1007/s10198-005-0276-3 15761775

[25] Sabale U, Ekman M, Granstrom O, Bergenheim K, McEwan P. Cost-effectiveness of dapagliflozin (Forxiga(R)) added to metformin compared with sulfonylurea added to metformin in type 2 diabetes in the Nordic countries. Prim Care Diabetes. 2015; 9: 39–47. 10.1016/j.pcd.2014.04.007 24840612

[26] Vivian EM. Dapagliflozin: a new sodium-glucose cotransporter 2 inhibitor for treatment of type 2 diabetes. Am J Health Syst Pharm. 2015; 72: 361–372. 10.2146/ajhp140168 25694411

[27] Komoroski B, Vachharajani N, Feng Y, Li L, Kornhauser D, Pfister M. Dapagliflozin, a novel, selective SGLT2 inhibitor, improved glycemic control over 2 weeks in patients with type 2 diabetes mellitus. Clin Pharmacol Ther. 2009; 85: 513–519. 10.1038/clpt.2008.250 19129749

[28] Parikh S, Wilding J, Jabbour S, Hardy E. Dapagliflozin in type 2 diabetes: effectiveness across the spectrum of disease and over time. Int J Clin Pract. 2015; 69: 186–198. 10.1111/ijcp.12531 25438821

[29] Bailey CJ, Morales VE, Woo V, Tang W, Ptaszynska A, List JF. Efficacy and safety of dapagliflozin monotherapy in people with Type 2 diabetes: a randomized double-blind placebo-controlled 102-week trial. Diabet Med. 2015; 32: 531–541. 10.1111/dme.12624 25381876

[30] Bailey CJ, Gross JL, Pieters A, Bastien A, List JF. Effect of dapagliflozin in patients with type 2 diabetes who have inadequate glycaemic control with metformin: a randomised, double-blind, placebo-controlled trial. Lancet. 2010; 375: 2223–2233. 10.1016/S0140-6736(10)60407-2 20609968

[31] Strojek K, Yoon KH, Hruba V, Elze M, Langkilde AM, Parikh S. Effect of dapagliflozin in patients with type 2 diabetes who have inadequate glycaemic control with glimepiride: a randomized, 24-week, double-blind, placebo-controlled trial. Diabetes Obes Metab. 2011; 13: 928–938. 10.1111/j.1463-1326.2011.01434.x 21672123

[32] Wilding JP, Norwood P, T'Joen C, Bastien A, List JF, Fiedorek FT. A study of dapagliflozin in patients with type 2 diabetes receiving high doses of insulin plus insulin sensitizers: applicability of a novel insulin-independent treatment. Diabetes Care. 2009; 32: 1656–1662. 10.2337/dc09-0517 19528367 PMC2732143

[33] Ferrannini E, Solini A. SGLT2 inhibition in diabetes mellitus: rationale and clinical prospects. Nat Rev Endocrinol. 2012; 8: 495–502. 10.1038/nrendo.2011.243 22310849

[34] Bailey CJ, Day C. SGLT2 inhibitors: glucuretic treatment for type 2 diabetes. The British Journal of Diabetes \& Vascular Disease. 2010; 10: 193–199.

[35] Shi J, Fang Z, Lin Y. Meta-analysis on effects of dapagliflozin on hyperglycaemia and cardiovascular risk factors for patients with type 2 diabetes. The Chinese Journal of Clinical Pharmacology. 2015; 31: 116–118, 134.

[36] Fioretto P, Giaccari A, Sesti G. Efficacy and safety of dapagliflozin, a sodium glucose cotransporter 2 (SGLT2) inhibitor, in diabetes mellitus. Cardiovasc Diabetol. 2015; 14: 142 10.1186/s12933-015-0297-x 26474563 PMC4609166

[37] Ptaszynska A, Johnsson KM, Parikh SJ, de Bruin T, Apanovitch AM, List JF. Safety Profile of Dapagliflozin for Type 2 Diabetes: Pooled Analysis of Clinical Studies for Overall Safety and Rare Events. DRUG SAFETY. 2014; 37: 815–829. 10.1007/s40264-014-0213-4 25096959

[38] Henry RR, Murray AV, Marmolejo MH, Hennicken D, Ptaszynska A, List JF. Dapagliflozin, metformin XR, or both: initial pharmacotherapy for type 2 diabetes, a randomised controlled trial. International Journal of Clinical Practice. 2012; 66: 446–456. 10.1111/j.1742-1241.2012.02911.x 22413962

[39] Ferrannini E, Ramos SJ, Salsali A, Tang W, List JF. Dapagliflozin monotherapy in type 2 diabetic patients with inadequate glycemic control by diet and exercise: a randomized, double-blind, placebo-controlled, phase 3 trial. Diabetes Care. 2010; 33: 2217–2224. 10.2337/dc10-0612 20566676 PMC2945163

[40] List JF, Woo V, Morales E, Tang W, Fiedorek FT. Sodium-glucose cotransport inhibition with dapagliflozin in type 2 diabetes. Diabetes Care. 2009; 32: 650–657. 10.2337/dc08-1863 19114612 PMC2660449

[41] Kaku K, Inoue S, Matsuoka O, Kiyosue A, Azuma H, Hayashi N, et al Efficacy and safety of dapagliflozin as a monotherapy for type 2 diabetes mellitus in Japanese patients with inadequate glycaemic control: a phase II multicentre, randomized, double-blind, placebo-controlled trial. Diabetes Obes Metab. 2013; 15: 432–440. 10.1111/dom.12047 23194084

[42] Ji L, Ma J, Li H, Mansfield TA, T'Joen CL, Iqbal N, et al Dapagliflozin as monotherapy in drug-naive Asian patients with type 2 diabetes mellitus: a randomized, blinded, prospective phase III study. Clin Ther. 2014; 36: 84–100. 10.1016/j.clinthera.2013.11.002 24378206

[43] Kaku K, Kiyosue A, Inoue S, Ueda N, Tokudome T, Yang J, et al Efficacy and safety of dapagliflozin monotherapy in Japanese patients with type 2 diabetes inadequately controlled by diet and exercise. Diabetes Obes Metab. 2014; 16: 1102–1110. 10.1111/dom.12325 24909293

[44] AstraZeneca. Forxiga 5 mg & 10 mg film coated tablets- 4. Clinical particulars. Available at http://www.medicines.org.uk/emc/medicine/27188/SPC/Forxiga+5mg++%26+10+mg+film+coated+tablets/. Accessed January 22, 2016.

[45] Ji L, Lu J, Weng J, Jia W, Tian H, Zhu D, et al China type 2 diabetes treatment status survey of treatment pattern of oral drugs users. J Diabetes. 2015; 7: 166–173. 10.1111/1753-0407.12165 24809622

[46] Gu S, Shi J, Tang Z, Sawhney M, Hu H, Shi L, et al Comparison of glucose lowering effect of metformin and acarbose in type 2 diabetes mellitus: a meta-analysis. PLoS One. 2015; 10: e126704. 10.1371/journal.pone.0126704 PMC4427275 25961824

[47] Yang W, Liu J, Shan Z, Tian H, Zhou Z, Ji Q, et al Acarbose compared with metformin as initial therapy in patients with newly diagnosed type 2 diabetes: an open-label, non-inferiority randomised trial. Lancet Diabetes Endocrinol. 2014; 2: 46–55. 10.1016/S2213-8587(13)70021-4 24622668

[48] Gu S, Tang Z, Shi L, Sawhney M, Hu H, Dong H. Cost-Minimization Analysis of Metformin and Acarbose in Treatment of Type 2 Diabetes. Value in Health Regional Issues. 2015; 6: 84–88. 29698199 10.1016/j.vhri.2015.03.012

[49] McEwan P, Peters JR, Bergenheim K, Currie CJ. Evaluation of the costs and outcomes from changes in risk factors in type 2 diabetes using the Cardiff stochastic simulation cost-utility model (DiabForecaster). Curr Med Res Opin. 2006; 22: 121–129. 10.1185/030079906X80350 16393438

[50] McEwan P, Evans M, Bergenheim K. A population model evaluating the costs and benefits associated with different oral treatment strategies in people with type 2 diabetes. Diabetes Obes Metab. 2010; 12: 623–630. 10.1111/j.1463-1326.2010.01198.x 20590737

[51] McEwan P, Evans M, Kan H, Bergenheim K. Understanding the inter-relationship between improved glycaemic control, hypoglycaemia and weight change within a long-term economic model. Diabetes Obes Metab. 2010; 12: 431–436. 10.1111/j.1463-1326.2009.01184.x 20415691

[52] McEwan P, Bergenheim K, Yuan Y, Tetlow AP, Gordon JP. Assessing the relationship between computational speed and precision: a case study comparing an interpreted versus compiled programming language using a stochastic simulation model in diabetes care. Pharmacoeconomics. 2010; 28: 665–674. 10.2165/11535350-000000000-00000 20524723

[53] Clarke PM, Gray AM, Briggs A, Farmer AJ, Fenn P, Stevens RJ, et al A model to estimate the lifetime health outcomes of patients with type 2 diabetes: the United Kingdom Prospective Diabetes Study (UKPDS) Outcomes Model (UKPDS no. 68). Diabetologia. 2004; 47: 1747–1759. 10.1007/s00125-004-1527-z 15517152

[54] World Health Organization. The world health report 2002- Chapter 5 (Some Strategies to Reduce Risk- Technical considerations for cost-effectiveness analysis). http://www.who.int/whr/2002/en/Chapter5.pdf?ua=1. Accessed January 24, 2016.

[55] Hotta N, Kakuta H, Sano T, Matsumae H, Yamada H, Kitazawa S, et al Long-term effect of acarbose on glycaemic control in non-insulin-dependent diabetes mellitus: a placebo-controlled double-blind study. Diabet Med. 1993; 10: 134–138. 8458189 10.1111/j.1464-5491.1993.tb00030.x

[56] Chan JC, Chan KW, Ho LL, Fuh MM, Horn LC, Sheaves R, et al An Asian multicenter clinical trial to assess the efficacy and tolerability of acarbose compared with placebo in type 2 diabetic patients previously treated with diet. Asian Acarbose Study Group. Diabetes Care. 1998; 21: 1058–1061. 9653595 10.2337/diacare.21.7.1058

[57] The Cochrane Collaboration. Cochrane handbook for systematic reviews of interventions. Available: http://www.cochrane-handbook.org. Accessed January 23, 2016.

[58] Zhu Q, Tong Y, Wu T, Li J, Tong N. Comparison of the hypoglycemic effect of acarbose monotherapy in patients with type 2 diabetes mellitus consuming an Eastern or Western diet: a systematic meta-analysis. Clin Ther. 2013; 35: 880–899. 10.1016/j.clinthera.2013.03.020 23602502

[59] Van de Laar F, Lucassen P, Akkermans R, Van de Lisdonk E, Rutten G, Van Weel C (2009) Alpha-glucosidase inhibitors for type 2 diabetes mellitus (Review). pp. 1–180. 10.1002/14651858.CD003639.pub2 PMC8406605 15846673

[60] Bucher HC, Guyatt GH, Griffith LE, Walter SD. The results of direct and indirect treatment comparisons in meta-analysis of randomized controlled trials. J Clin Epidemiol. 1997; 50: 683–691. 9250266 10.1016/s0895-4356(97)00049-8

[61] Glenny AM, Altman DG, Song F, Sakarovitch C, Deeks JJ, D'Amico R, et al Indirect comparisons of competing interventions. Health Technol Assess. 2005; 9: 1–134. 10.3310/hta9260 16014203

[62] Wells GA, Sultan SA, Chen L, Khan M, Coyle D Indirect evidence: Indirect treatment comparisons in meta-analysis Ottawa: Canadian Agency for Drugs and Technologies in Health 2009.

[63] Song F, Altman DG, Glenny AM, Deeks JJ. Validity of indirect comparison for estimating efficacy of competing interventions: empirical evidence from published meta-analyses. BMJ. 2003; 326: 472 10.1136/bmj.326.7387.472 12609941 PMC150178

[64] General Administration Of Sport Of China. National Physique Monitoring Bulletin (2014). http://www.sport.gov.cn/n16/n1077/n1227/7328132.html. Accessed January 24, 2016.

[65] Worldwide Inflation Data. Average inflation China (CPI). http://www.inflation.eu/inflation-rates/china/historic-inflation/cpi-inflation-china.aspxAccessed March 4, 2016.

[66] The Organisation for Economic Co-operation and Development (OECD). Exchange rates. https://data.oecd.org/conversion/exchange-rates.htm. Accessed October 6, 2016.

[67] Gao L, Zhao FL, Li SC. Cost-utility analysis of liraglutide versus glimepiride as add-on to metformin in type 2 diabetes patients in China. Int J Technol Assess Health Care. 2012; 28: 436–444. 10.1017/S0266462312000608 23006540

[68] Li H, Xu F, Wang F. Cost-effectiveness of biphasic insulin aspart 30 combined with metformin in patients with type 2 diabetes mellitus. Chinese Journal New Drugs. 2011; 20: 2163–2170.

[69] Palmer JL, Gibbs M, Scheijbeler HW, Kotchie RW, Nielsen S, White J, et al Cost-effectiveness of switching to biphasic insulin aspart in poorly-controlled type 2 diabetes patients in China. Adv Ther. 2008; 25: 752–774. 10.1007/s12325-008-0080-4 18704282

[70] Zheng Y, Wu J, Xie K. Incidence and cost of hypoglycemia episode in patients with type 2 diabetes mellitus (T2DM). Chinese Rural Health Service Administration. 2012; 32: 1195–1198.

[71] Meng H. Pharmacoeconomic analysis of the treatment of type 2 diabetes complicated with urinary infections with two different antibiotics. JOURNAL OF SHANDONG MIEICAL COLLEGE. 2009; 31: 91–93.

[72] Marrett E, Radican L, Davies MJ, Zhang Q. Assessment of severity and frequency of self-reported hypoglycemia on quality of life in patients with type 2 diabetes treated with oral antihyperglycemic agents: A survey study. BMC Res Notes. 2011; 4: 251 10.1186/1756-0500-4-251 21777428 PMC3148563

[73] Amiel SA, Dixon T, Mann R, Jameson K. Hypoglycaemia in Type 2 diabetes. Diabet Med. 2008; 25: 245–254. 10.1111/j.1464-5491.2007.02341.x 18215172 PMC2327221

[74] Guo H, Li J, Jiang Z. Follow-up effects of the increased physical activity on the glucolipid metabolic factors and medical costs in type 2 diabetic patients. CHINESE JOURNAL OF REHABILITATION MEDICINE. 2007; 22: 395–398.

[75] Price Bureau. Official drug price list. http://www.zjpi.gov.cn/main/html/2013/CT10138/75fbbde9c06b496aba6490646b9ca77f.html. Accessed January 27, 2016.

[76] Bank Of China BOC. Exchange Rate. http://www.boc.cn/sourcedb/whpj/. Accessed March 2, 2016.

[77] Clarke P, Gray A, Holman R. Estimating utility values for health states of type 2 diabetic patients using the EQ-5D (UKPDS 62). Med Decis Making. 2002; 22: 340–349. 12150599 10.1177/0272989X0202200412

[78] Currie CJ, McEwan P, Peters JR, Patel TC, Dixon S. The routine collation of health outcomes data from hospital treated subjects in the Health Outcomes Data Repository (HODaR): descriptive analysis from the first 20,000 subjects. Value Health. 2005; 8: 581–590. 10.1111/j.1524-4733.2005.00046.x 16176496

[79] Currie CJ, Morgan CL, Poole CD, Sharplin P, Lammert M, McEwan P. Multivariate models of health-related utility and the fear of hypoglycaemia in people with diabetes. Curr Med Res Opin. 2006; 22: 1523–1534. 10.1185/030079906X115757 16870077

[80] Lane S, Levy AR, Mukherjee J, Sambrook J, Tildesley H. The impact on utilities of differences in body weight among Canadian patients with type 2 diabetes. CURRENT MEDICAL RESEARCH AND OPINION. 2014; 30: 1267–1273. 24588550 10.1185/03007995.2014.899207

[81] Matza LS, Boye KS, Yurgin N, Brewster-Jordan J, Mannix S, Shorr JM, et al Utilities and disutilities for type 2 diabetes treatment-related attributes. Qual Life Res. 2007; 16: 1251–1265. 10.1007/s11136-007-9226-0 17638121

[82] Barry HC, Ebell MH, Hickner J. Evaluation of suspected urinary tract infection in ambulatory women: a cost-utility analysis of office-based strategies. J Fam Pract. 1997; 44: 49–60. 9010371

[83] Caro JJ, Stillman IO, Danel A, Getsios D, McEwan P. Cost effectiveness of rimonabant use in patients at increased cardiometabolic risk: estimates from a Markov model. Journal of Medical Economics. 2007; 10: 239–254.

[84] National Bureau of Statistics of China. Gross Domestic Product (GDP) per capita. http://data.stats.gov.cn/easyquery.htm?cn=C01&zb=A0201&sj=2014. Accessed January 28, 2016.

[85] Lee J, Ma S, Heng D, Chew S, Hughes K, Tai E. Hypertension, concurrent cardiovascular risk factors and mortality: the Singapore Cardiovascular Cohort Study. J Hum Hypertens. 2008; 22: 468–474. 10.1038/jhh.2008.16 18337755

[86] Chen G, McAlister FA, Walker RL, Hemmelgarn BR, Campbell NR. Cardiovascular outcomes in framingham participants with diabetes: the importance of blood pressure. Hypertension. 2011; 57: 891–897. 10.1161/HYPERTENSIONAHA.110.162446 21403089 PMC3785072

[87] Peters AL. Patient and treatment perspectives: Revisiting the link between type 2 diabetes, weight gain, and cardiovascular risk. Cleve Clin J Med. 2009; 76 Suppl 5: S20–S27. 10.3949/ccjm.76.s5.04 19952300

[88] Bodegard J, Sundstrom J, Svennblad B, Ostgren CJ, Nilsson PM, Johansson G. Changes in body mass index following newly diagnosed type 2 diabetes and risk of cardiovascular mortality: a cohort study of 8486 primary-care patients. Diabetes Metab. 2013; 39: 306–313. 10.1016/j.diabet.2013.05.004 23871502

[89] Bolin K, Gip C, Mork AC, Lindgren B. Diabetes, healthcare cost and loss of productivity in Sweden 1987 and 2005—a register-based approach. Diabet Med. 2009; 26: 928–934. 10.1111/j.1464-5491.2009.02786.x 19719715

[90] Yu AP, Wu EQ, Birnbaum HG, Emani S, Fay M, Pohl G, et al Short-term economic impact of body weight change among patients with type 2 diabetes treated with antidiabetic agents: analysis using claims, laboratory, and medical record data. CURRENT MEDICAL RESEARCH AND OPINION. 2007; 23: 2157–2169. 17669232 10.1185/0300799007X219544

[91] Wing RR, Lang W, Wadden TA, Safford M, Knowler WC, Bertoni AG, et al Benefits of modest weight loss in improving cardiovascular risk factors in overweight and obese individuals with type 2 diabetes. Diabetes Care. 2011; 34: 1481–1486. 10.2337/dc10-2415 21593294 PMC3120182

[92] Zullig LL, Gellad WF, Moaddeb J, Crowley MJ, Shrank W, Granger BB, et al Improving diabetes medication adherence: successful, scalable interventions. Patient Prefer Adherence. 2015; 9: 139–149. 10.2147/PPA.S69651 25670885 PMC4315534

[93] Ho PM, Rumsfeld JS, Masoudi FA, McClure DL, Plomondon ME, Steiner JF, et al Effect of medication nonadherence on hospitalization and mortality among patients with diabetes mellitus. ARCHIVES OF INTERNAL MEDICINE. 2006; 166: 1836–1841. 10.1001/archinte.166.17.1836 17000939

[94] White TJ, Vanderplas A, Chang E, Dezii CM, Abrams GD. The costs of non-adherence to oral antihyperglycemic medication in individuals with diabetes Mellitus and concomitant diabetes mellitus and cardiovascular disease in a managed care environment. DISEASE MANAGEMENT & HEALTH OUTCOMES. 2004; 12: 181–188.

[95] Currie CJ, Peyrot M, Morgan CL, Poole CD, Jenkins-Jones S, Rubin RR, et al The impact of treatment noncompliance on mortality in people with type 2 diabetes. Diabetes Care. 2012; 35: 1279–1284. 10.2337/dc11-1277 22511257 PMC3357221

[96] Miller BR, Nguyen H, Hu CJ, Lin C, Nguyen QT. New and emerging drugs and targets for type 2 diabetes: reviewing the evidence. Am Health Drug Benefits. 2014; 7: 452–463. 25558307 PMC4280522

[97] Tunceli K, Zhao C, Davies MJ, Brodovicz KG, Alexander CM, Iglay K, et al Factors associated with adherence to oral antihyperglycemic monotherapy in patients with type 2 diabetes. Patient Prefer Adherence. 2015; 9: 191–197. 10.2147/PPA.S71346 25670888 PMC4315552

[98] Donnan PT, MacDonald TM, Morris AD. Adherence to prescribed oral hypoglycaemic medication in a population of patients with Type 2 diabetes: a retrospective cohort study. Diabet Med. 2002; 19: 279–284. 11942998 10.1046/j.1464-5491.2002.00689.x

[99] Bayer. Glucobay 50mg tablets- 4. Clinical particulars. Available at http://www.medicines.org.uk/emc/medicine/19972. Accessed February 8, 2016.

[100] Charokopou M, McEwan P, Lister S, Callan L, Bergenheim K, Tolley K, et al Cost-effectiveness of dapagliflozin versus DPP-4 inhibitors as an add-on to Metformin in the Treatment of Type 2 Diabetes Mellitus from a UK Healthcare System Perspective. BMC Health Serv Res. 2015; 15: 496 10.1186/s12913-015-1139-y 26541516 PMC4635987

[101] Abad PE, Casado EP, Fernandez RJ, Morales EF, Betegon NL, Sanchez-Covisa J, et al Cost-effectiveness analysis of dapagliflozin compared to DPP4 inhibitors and other oral antidiabetic drugs in the treatment of type-2 diabetes mellitus in Spain. Aten Primaria. 2015; 47: 505–513. 10.1016/j.aprim.2014.11.002 25555492 PMC6983792

[102] Charokopou M, McEwan P, Lister S, Callan L, Bergenheim K, Tolley K, et al The cost-effectiveness of dapagliflozin versus sulfonylurea as an add-on to metformin in the treatment of Type 2 diabetes mellitus. Diabet Med. 2015; 32: 890–898. 10.1111/dme.12772 25817050

[103] van Haalen HG, Pompen M, Bergenheim K, McEwan P, Townsend R, Roudaut M. Cost effectiveness of adding dapagliflozin to insulin for the treatment of type 2 diabetes mellitus in the Netherlands. Clin Drug Investig. 2014; 34: 135–146. 10.1007/s40261-013-0155-0 24243529

