# Supplementary material for: Cost-Effectiveness of Dapagliflozin versus Acarbose as a Monotherapy in Type 2 Diabetes in China
Source: PLoS One. 2016 Nov 2;11(11):e0165629. doi: 10.1371/journal.pone.0165629 (PMC5091768; doi:10.1371/journal.pone.0165629)
Supplement: S1 Appendix — (PDF) [file pone.0165629.s002.pdf]

S2: Selection Criteria

Inclusion criteria:

(1) Only randomized controlled trials were eligible;

(2) Head-to-head studies compared clinical effect of dapagliflozin versus placebo (or acarbose versus placebo);

(3) Studies on Asian patients with type 2 Diabetes (T2DM) who are 18 years or older;

(4) Patients treated with monotherapy, and only diet or/and exercise were allowed;

(5) Studies written in English;

(6) Studies had glycosylated hemoglobin (HbA_{1c}), systolic blood pressure (SBP), body weights, total cholesterol, high density lipoprotein (HDL) cholesterol, or hypoglycemia as outcome indicator;

(7) Studies with a follow-up duration of 12 weeks or longer;

(8) Studies focused on patients whose estimated glomerular filtration rate (eGFR) is over 60 mL/min/1.73 m^2.

Exclusion criteria:

(1) Studies not related to or not targeted at dapagliflozin (or acarbose), including studies merely on sodium glucose co-transporter 2 inhibitors (SGLT2), or alpha-glucosidase inhibitors;

(2) Studies only provided information about dapagliflozin (or acarbose), including introduction of pharmacokinetics, pharmacologic properties, pharmacodynamics, clinical use, safety, tolerability or mechanism of action;

(3) Studies not targeted at T2DM, including impaired glucose tolerance (IGT), impaired fasting glucose (IFG), gestational diabetes or type 1 diabetes (T1DM);

(4) Studies on cell lines or animals;

(5) Literature reviews or meta-analysis;

(6) Meeting abstracts;

(7) Economic evaluation studies;

(8) Letters or comments;

(9) Studies were not monotherapy design;

(10) Studies included patients whose eGFR is below 60 mL/min/1.73 m^2.
