# Supplementary figures and images for: Cost-Effectiveness of Dapagliflozin versus Acarbose as a Monotherapy in Type 2 Diabetes in China
Source: PLoS One. 2016 Nov 2;11(11):e0165629. doi: 10.1371/journal.pone.0165629 (PMC5091768; doi:10.1371/journal.pone.0165629)

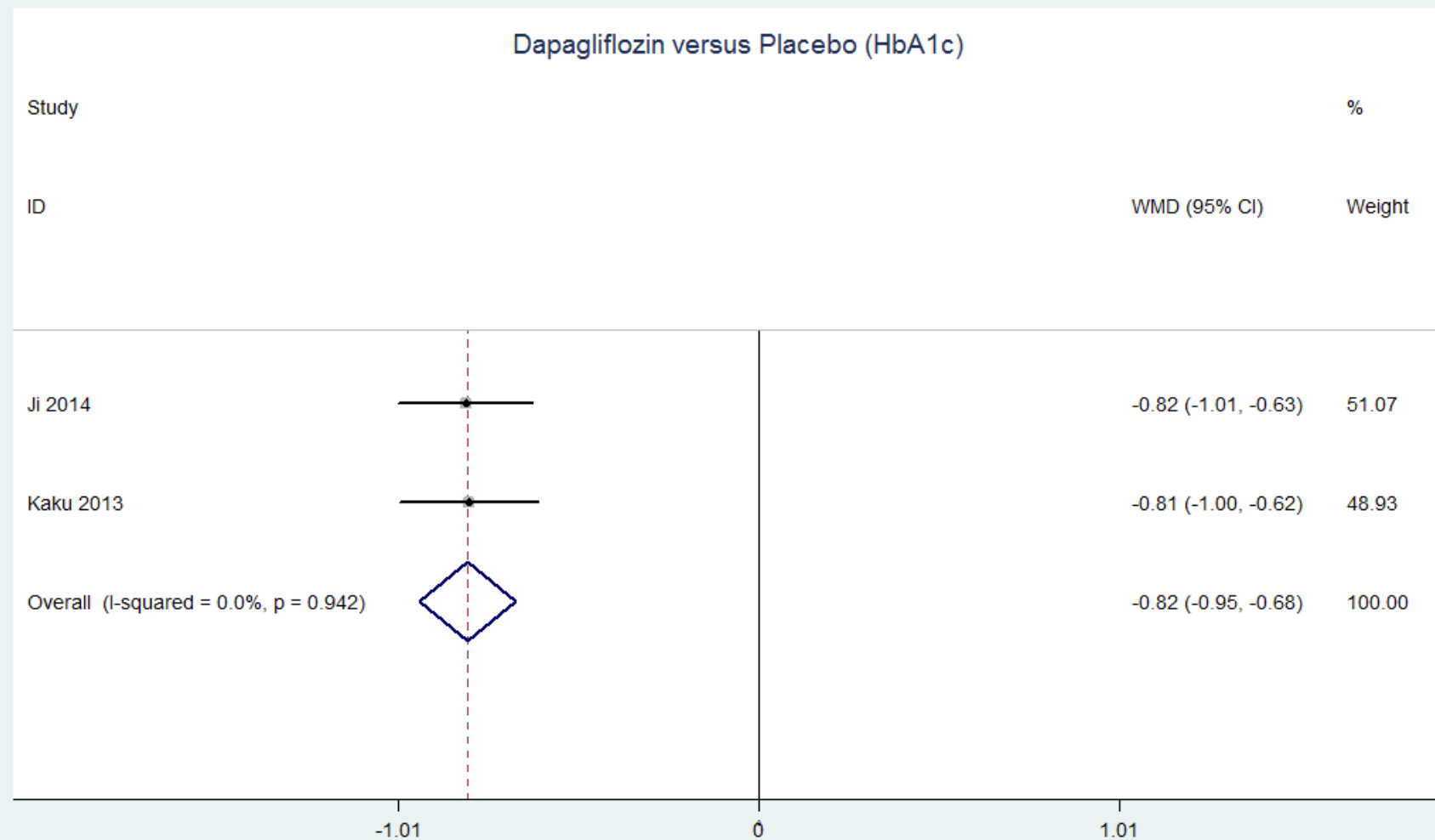

**S1 Fig. Meta-analysis of dapagliflozin versus placebo on HbA1c**

Supplement: S1 Fig — (PDF) [file pone.0165629.s003.pdf]

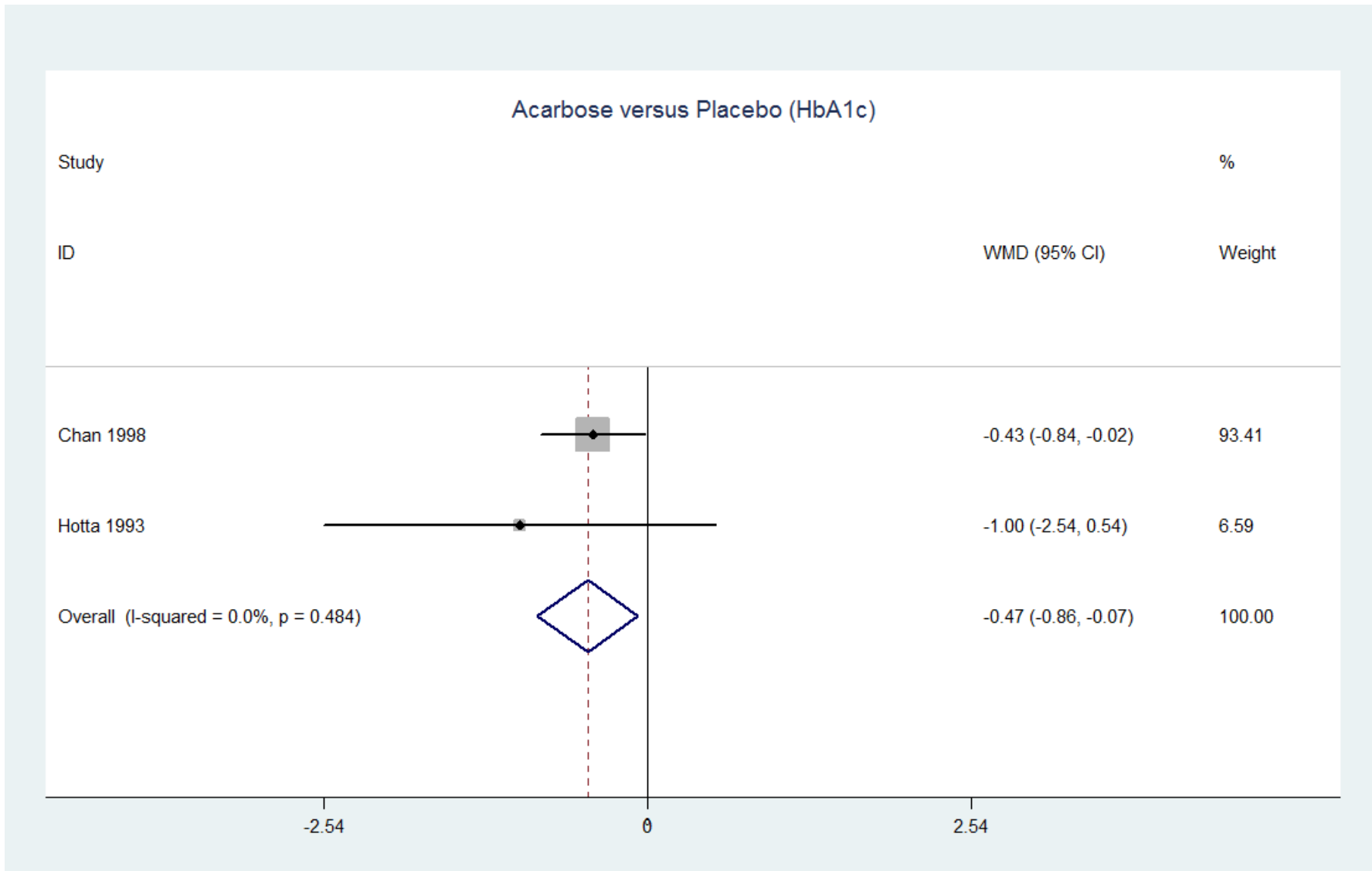

S2 Fig. Meta-analysis of acarbose versus placebo on HbA1c

Supplement: S2 Fig — (PDF) [file pone.0165629.s004.pdf]

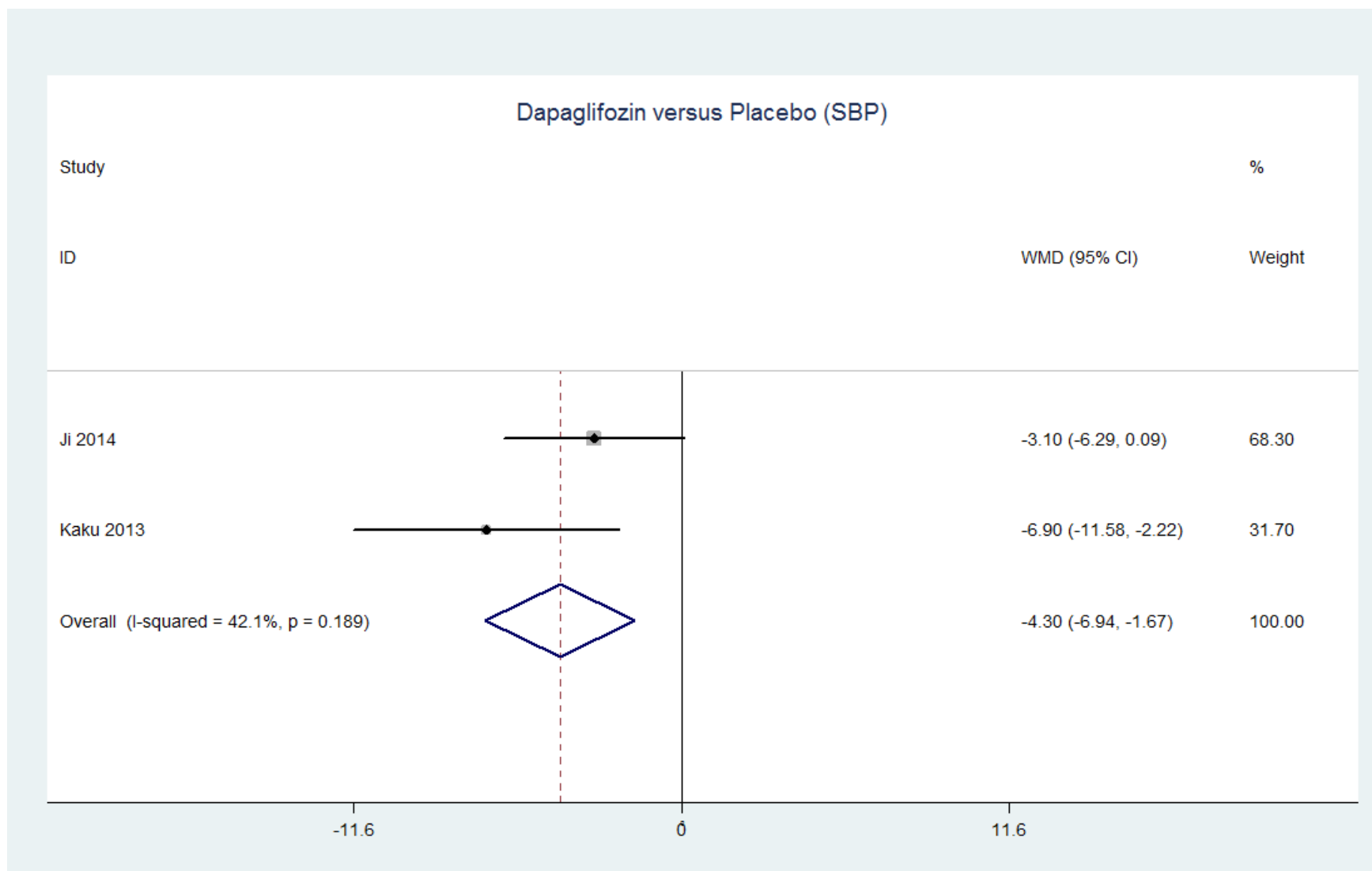

**S3 Fig. Meta-analysis of dapagliflozin versus placebo on SBP**

Supplement: S3 Fig — (PDF) [file pone.0165629.s005.pdf]

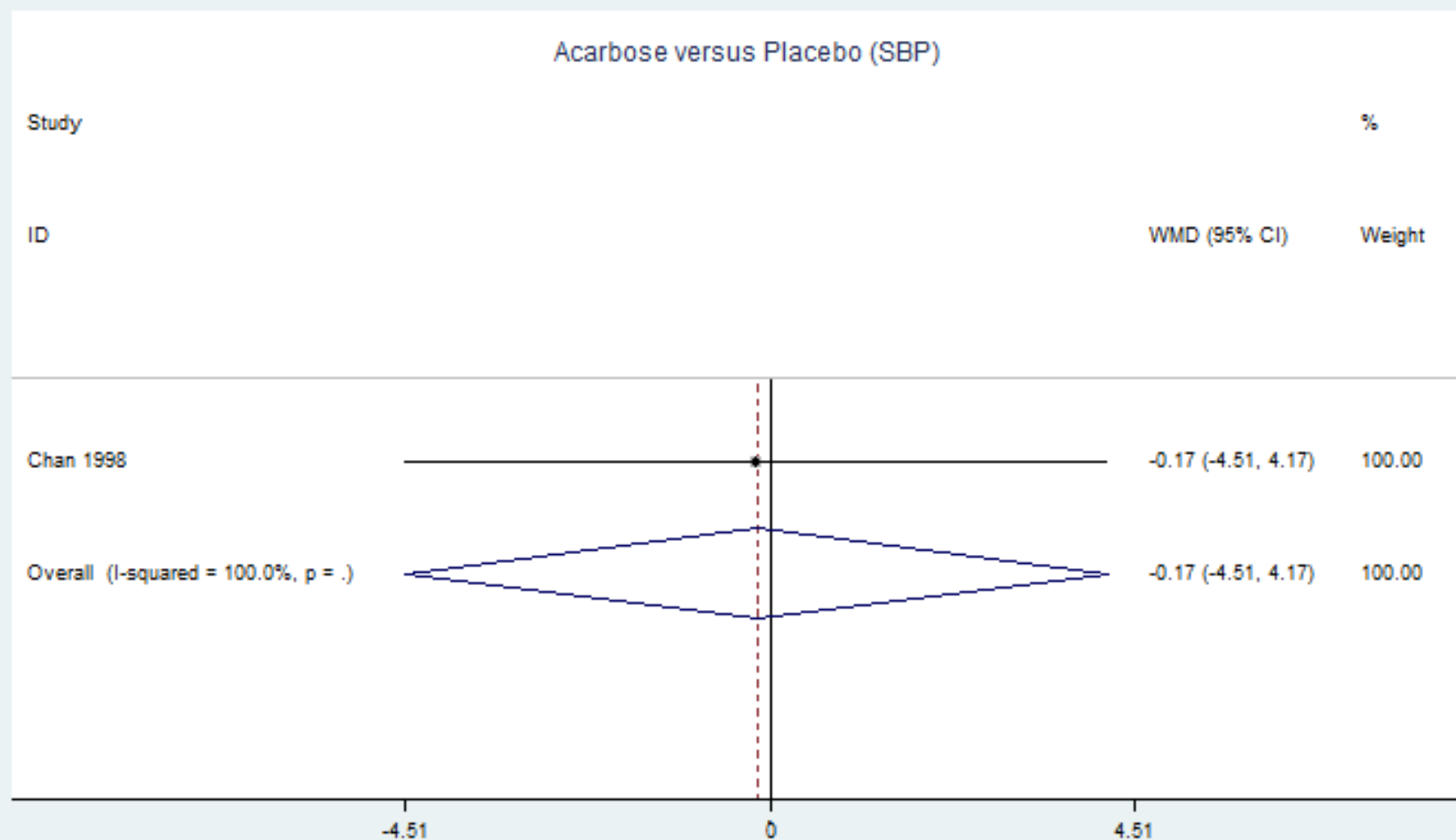

**S4 Fig. Meta-analysis of acarbose versus placebo on SBP**

Supplement: S4 Fig — (PDF) [file pone.0165629.s006.pdf]

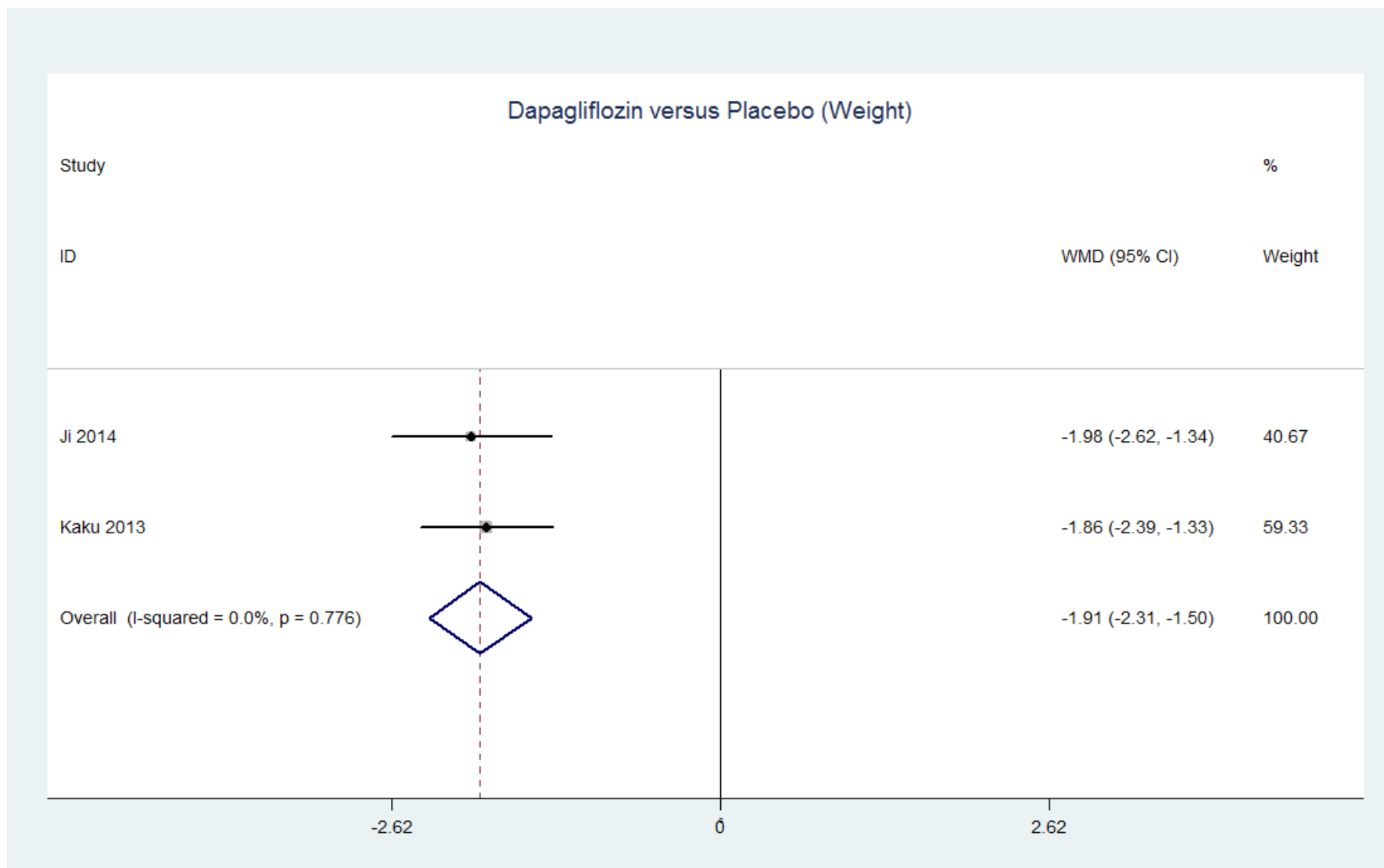

**S5 Fig. Meta-analysis of dapagliflozin versus placebo on weight**

Supplement: S5 Fig — (PDF) [file pone.0165629.s007.pdf]

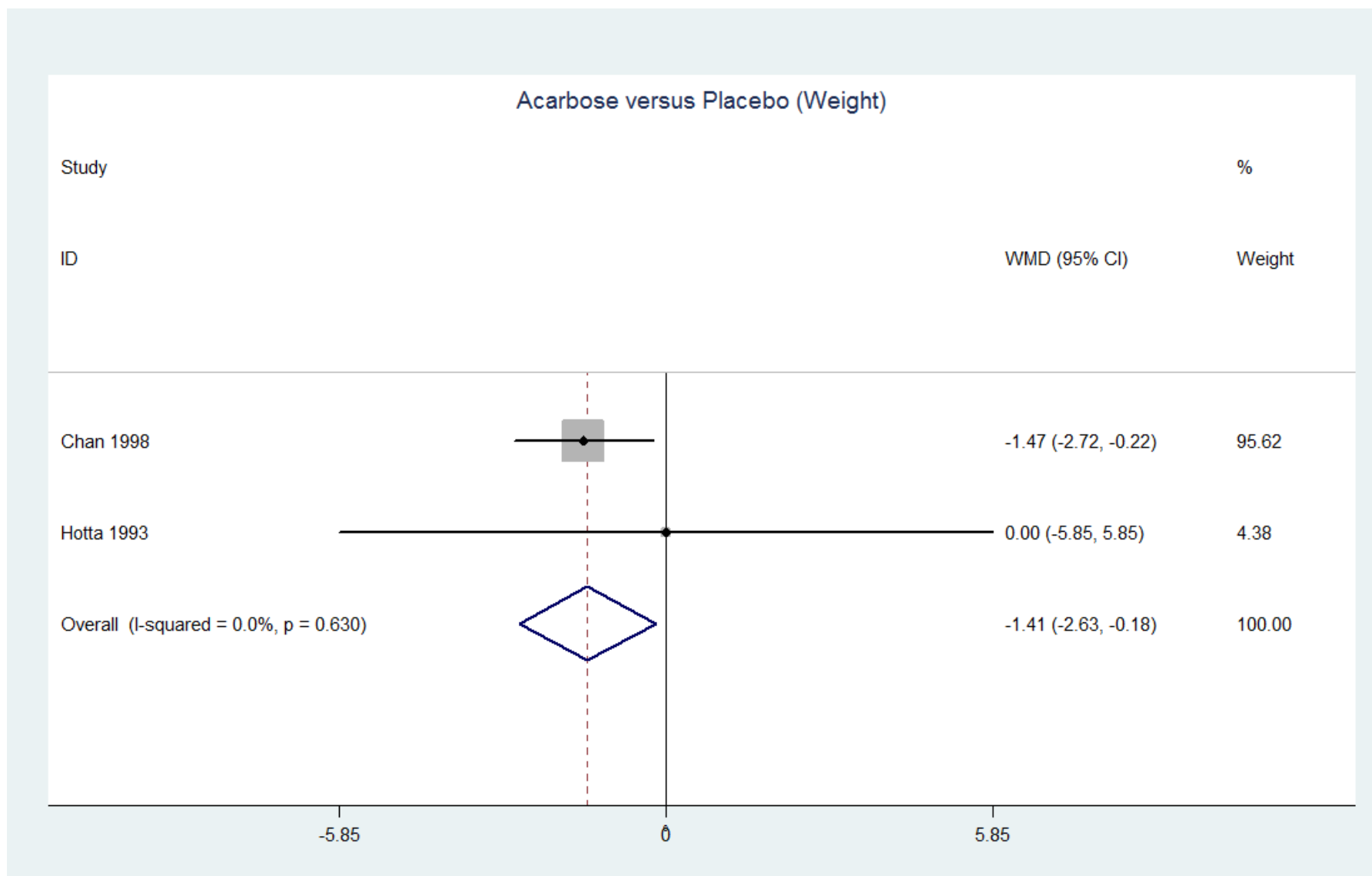

**S6 Fig. Meta-analysis of acarbose versus placebo on weight**

Supplement: S6 Fig — (PDF) [file pone.0165629.s008.pdf]
