# Supplementary material for: Cost-Effectiveness of Dapagliflozin versus Acarbose as a Monotherapy in Type 2 Diabetes in China
Source: PLoS One. 2016 Nov 2;11(11):e0165629. doi: 10.1371/journal.pone.0165629 (PMC5091768; doi:10.1371/journal.pone.0165629)
Supplement: S1 Table — (PDF) [file pone.0165629.s009.pdf]

S1 Table. Search strategy

Database 1: PubMed

Keywords	Query	Items found
**Part 1: Dapagliflozin**		
#1	Search "2-(3-(4-ethoxybenzyl)-4-chlorophenyl)-6-hydroxymethyltetrahydro-2H-pyran-3,4,5-triol" [Supplementary Concept]	184
#2	**#1 OR (dapagliflozin):**<br>Search (dapagliflozin) OR "2-(3-(4-ethoxybenzyl)-4-chlorophenyl)-6-hydroxymethyltetrahydro-2H-pyran-3,4,5-triol" [Supplementary Concept]	339
**Part 2: Acarbose**		
#3	Search "Acarbose"[Mesh] OR "acarbose byproduct, component C" [Supplementary Concept] OR "acarbose 7-phosphotransferase" [Supplementary Concept] OR "acarbose 7-phosphate" [Supplementary Concept]	1137
#4	**#3 OR (acarbose):**<br>Search (acarbose) OR ("Acarbose"[Mesh] OR "acarbose byproduct, component C" [Supplementary Concept] OR "acarbose 7-phosphotransferase" [Supplementary Concept] OR "acarbose 7-phosphate" [Supplementary Concept])	1935
**Part 3: Placebo**		
#5	Search "Placebos"[Mesh] OR "Placebo Effect"[Mesh]	35817
#6	**#5 OR (placebo):**<br>Search (placebo) OR ("Placebos"[Mesh] OR "Placebo Effect"[Mesh])	186754
**Part 4: Type 2 diabetes**		
#7	Search "Diabetes Mellitus, Type 2"[Mesh]	94459
#8	**#7 OR (type 2 diabetes):**<br>Search (type 2 diabetes) OR "Diabetes Mellitus, Type 2"[Mesh]	124694
**Part 5: (Dapagliflozin) OR (Acarbose)**		
#9	**#2 OR #4:**<br>Search (((dapagliflozin) OR "2-(3-(4-ethoxybenzyl)-4-chlorophenyl)-6-hydroxymethyltetrahydro-2H-pyran-3,4,5-triol" [Supplementary Concept])) OR ((acarbose) OR ("Acarbose"[Mesh] OR "acarbose byproduct, component C" [Supplementary Concept] OR "acarbose 7-phosphotransferase" [Supplementary Concept] OR "acarbose 7-phosphate" [Supplementary Concept]))	2274

Part 6: ((Dapagliflozin) OR (Acarbose)) AND (Placebo)

#10	**#9 AND #6:**	291
	Search (((((dapagliflozin) OR "2-(3-(4-ethoxybenzyl)-4-chlorophenyl)-6-hydroxymethyltetrahydro-2H-pyran-3,4,5-triol" [Supplementary Concept])) OR ((acarbose) OR ("Acarbose"[Mesh] OR "acarbose byproduct, component C" [Supplementary Concept] OR "acarbose 7-phosphotransferase" [Supplementary Concept] OR "acarbose 7-phosphate" [Supplementary Concept])))) AND ((placebo) OR ("Placebos"[Mesh] OR "Placebo Effect"[Mesh]))	

Part 7: ((Dapagliflozin) OR (Acarbose)) AND (Placebo) AND (Type 2 diabetes)

#11	**#10 AND #8:**	218
	Search (((((((dapagliflozin) OR "2-(3-(4-ethoxybenzyl)-4-chlorophenyl)-6-hydroxymethyltetrahydro-2H-pyran-3,4,5-triol" [Supplementary Concept])) OR ((acarbose) OR ("Acarbose"[Mesh] OR "acarbose byproduct, component C" [Supplementary Concept] OR "acarbose 7-phosphotransferase" [Supplementary Concept] OR "acarbose 7-phosphate" [Supplementary Concept])))) AND ((placebo) OR ("Placebos"[Mesh] OR "Placebo Effect"[Mesh])))) AND ((type 2 diabetes) OR "Diabetes Mellitus, Type 2"[Mesh])	
#12	**#11 AND (1990/01/01- 2015/12/31[Date - Publication]):**	211
	Search (((((((((dapagliflozin) OR "2-(3-(4-ethoxybenzyl)-4-chlorophenyl)-6-hydroxymethyltetrahydro-2H-pyran-3,4,5-triol" [Supplementary Concept])) OR ((acarbose) OR ("Acarbose"[Mesh] OR "acarbose byproduct, component C" [Supplementary Concept] OR "acarbose 7-phosphotransferase" [Supplementary Concept] OR "acarbose 7-phosphate" [Supplementary Concept])))) AND ((placebo) OR ("Placebos"[Mesh] OR "Placebo Effect"[Mesh])))) AND ((type 2 diabetes) OR "Diabetes Mellitus, Type 2"[Mesh]))) AND ("1990/01/01"[Date - Publication] : "2015/12/31"[Date - Publication])	
#13	**#12 AND Filters: Humans:**	**180**
	Search (((((((((dapagliflozin) OR "2-(3-(4-ethoxybenzyl)-4-chlorophenyl)-6-hydroxymethyltetrahydro-2H-pyran-3,4,5-triol" [Supplementary Concept])) OR ((acarbose) OR ("Acarbose"[Mesh] OR "acarbose byproduct, component C" [Supplementary Concept] OR "acarbose 7-phosphotransferase" [Supplementary Concept] OR "acarbose 7-phosphate" [Supplementary Concept])))) AND ((placebo) OR ("Placebos"[Mesh] OR "Placebo Effect"[Mesh])))) AND ((type 2 diabetes) OR "Diabetes Mellitus, Type 2"[Mesh]))) AND ("1990/01/01"[Date - Publication] : "2015/12/31"[Date - Publication]) Filters: Humans	

Database 2: Web of Knowledge

Keywords	Query	Items found
#1	TS= dapagliflozin<br>*Time span= 1990-2015; Retrieval language =automatic*	816
#2	TS=acarbose<br>*Time span= 1990-2015; Retrieval language =automatic*	approximate 6,215
#3	TS= placebo<br>*Time span= 1990-2015; Retrieval language =automatic*	approximate 439,940
#4	TS=type 2 diabetes<br>*Time span= 1990-2015; Retrieval language =automatic*	approximate 399,014
#5	**#1 OR #2:**<br>(TS=dapagliflozin) OR (TS=acarbose)<br>*Time span= 1990-2015; Retrieval language =automatic*	approximate 7,515
#6	**#5 AND #3:**<br>((TS=dapagliflozin) OR (TS=acarbose)) AND (TS= placebo)<br>*Time span= 1990-2015; Retrieval language =automatic*	520
#7	**#6 AND (#4 OR TS=T2DM):**<br>((TS=dapagliflozin) OR (TS=acarbose)) AND (TS=placebo) AND ((TS=type 2 diabetes) OR (TS=T2DM))<br>*Time span= 1990-2015; Retrieval language =automatic*	**422**

Database 3: ScienceDirect

Expert search	Query	Items found
Search for	((title-abs-key (dapagliflozin) OR title-abs-key (acarbose)) AND (title-abs-key (placebo))) AND (title-abs-key (type 2 diabetes) OR title-abs-key (T2DM))	**27**
Refine your search	Journals; Books; All; -All Sciences-	
Years	1990-2015	

Database 4: OVID

#	Searches	Results	Search type
1	dapagliflozin {Including Limited Related Terms}	316	Basic
2	limit 1 to human [Limit not valid in Journals@Ovid, Your Journals@Ovid, CAB Abstracts, FSTA; records were retained]	304	Advanced
3	limit 2 to yr="1990 - 2015"	296	Advanced
4	acarbose {Including Limited Related Terms}	2699	Basic
5	limit 4 to human [Limit not valid in Journals@Ovid, Your Journals@Ovid, CAB Abstracts, FSTA; records were retained]	2197	Advanced
6	limit 5 to yr="1990 - 2015"	2170	Advanced
7	3 or 6	2463	Advanced
8	placebo {Including Limited Related Terms}	20584	Basic
9	limit 8 to human [Limit not valid in Journals@Ovid, Your Journals@Ovid, CAB Abstracts, FSTA; records were retained]	20288	Advanced
10	limit 9 to yr="1990 - 2015"	19990	Advanced
11	7 and 10	27	Advanced
12	type 2 diabetes {Including Limited Related Terms}	18967	Basic
13	limit 12 to human [Limit not valid in Journals@Ovid, Your Journals@Ovid, CAB Abstracts, FSTA; records were retained]	18364	Advanced
14	limit 13 to yr="1990 - 2015"	18251	Advanced
15	11 and 14	**4**	Advanced
