# Supplementary material for: Cost-Effectiveness of Dapagliflozin versus Acarbose as a Monotherapy in Type 2 Diabetes in China
Source: PLoS One. 2016 Nov 2;11(11):e0165629. doi: 10.1371/journal.pone.0165629 (PMC5091768; doi:10.1371/journal.pone.0165629)
Supplement: S2 Table — (PDF) [file pone.0165629.s010.pdf]

S2 Table. Characteristics of the included studies

Study	Study Design	Population	Basic Therapy	Study Duration	Invention	Sample size
Kaku 2013	A phase II, multicentre, randomized, five-arm, parallel-group, double-blind, placebo-controlled trial	Japan	diet and exercise	12 weeks	Dapagliflozin: 10mg/d; Placebo;	Dapagliflozin: 52; Placebo: 54;
Ji 2014	A phase III, multicentre, randomized, parallel-group, double-blind, placebo-controlled trial	China, Korea, Taiwan, India	diet and exercise	24 weeks	Dapagliflozin: 10mg/d; Placebo;	Dapagliflozin: 133; Placebo: 132;
Hotta 1993	A randomized, double-blind, placebo-controlled trial	Japan	diet	24 weeks	Acarbose: 300mg/d; Placebo;	Acarbose: 19; Placebo: 18;
Chan 1998	A randomized, double-blind multicentre trial	Taiwan, Hong Kong, Philippines, Korea, Singapore, Malaysia	diet	24 weeks	Acarbose: 300mg/d; Placebo;	Acarbose: 63; Placebo: 63;
