# Supplementary material for: Cost-Effectiveness of Dapagliflozin versus Acarbose as a Monotherapy in Type 2 Diabetes in China
Source: PLoS One. 2016 Nov 2;11(11):e0165629. doi: 10.1371/journal.pone.0165629 (PMC5091768; doi:10.1371/journal.pone.0165629)
Supplement: S3 Table — (PDF) [file pone.0165629.s011.pdf]

S3 Table. Quality of the included studies

Study	Selection Bias		Performance Bias	Attrition Bias	Overall Quality
	Randomization	Allocation Concealment	Blinding	Dropout/ loss to Follow-up	
Kaku 2013	A	A	A	A	A
Ji 2014	A	A	A	A	A
Hotta 1993	B	A	A	B	B
Chan 1998	B	B	A	A	B
